# Mining metadata from unidentified ITS sequences in GenBank: A case study in *Inocybe *(*Basidiomycota*)

**DOI:** 10.1186/1471-2148-8-50

**Published:** 2008-02-18

**Authors:** Martin Ryberg, R Henrik Nilsson, Erik Kristiansson, Mats Töpel, Stig Jacobsson, Ellen Larsson

**Affiliations:** 1 Department of Plant and Environmental Sciences, Göteborg University, Box 461, 405 30 Göteborg, Sweden; 2 Department of Mathematical Statistics, Chalmers University of Technology, Göteborg, Sweden

## Abstract

**Background:**

The lack of reference sequences from well-identified mycorrhizal fungi often poses a challenge to the inference of taxonomic affiliation of sequences from environmental samples, and many environmental sequences are thus left unidentified. Such unidentified sequences belonging to the widely distributed ectomycorrhizal fungal genus *Inocybe *(*Basidiomycota*) were retrieved from GenBank and divided into species that were identified in a phylogenetic context using a reference dataset from an ongoing study of the genus. The sequence metadata of the unidentified *Inocybe *sequences stored in GenBank, as well as data from the corresponding original papers, were compiled and used to explore the ecology and distribution of the genus. In addition, the relative occurrence of *Inocybe *was contrasted to that of other mycorrhizal genera.

**Results:**

Most species of *Inocybe *were found to have less than 3% intraspecific variability in the ITS2 region of the nuclear ribosomal DNA. This cut-off value was used jointly with phylogenetic analysis to delimit and identify unidentified *Inocybe *sequences to species level. A total of 177 unidentified *Inocybe *ITS sequences corresponding to 98 species were recovered, 32% of which were successfully identified to species level in this study. These sequences account for an unexpectedly large proportion of the publicly available unidentified fungal ITS sequences when compared with other mycorrhizal genera. Eight *Inocybe *species were reported from multiple hosts and some even from hosts forming arbutoid or orchid mycorrhizae. Furthermore, *Inocybe *sequences have been reported from four continents and in climate zones ranging from cold temperate to equatorial climate. Out of the 19 species found in more than one study, six were found in both Europe and North America and one was found in both Europe and Japan, indicating that at least many north temperate species have a wide distribution.

**Conclusion:**

Although DNA-based species identification and circumscription are associated with practical and conceptual difficulties, they also offer new possibilities and avenues for research. Metadata assembly holds great potential to synthesize valuable information from community studies for use in a species and taxonomy-oriented framework.

## Background

The taxonomy of fungi is largely based on ephemeral and irregularly occurring propagation structures, notably sexual fruiting-bodies. The main part of the fungal life cycle is, however, a somatic – typically mycelial – phase that offers few discriminatory morphological characters and that may be difficult to assign even to family or ordinal level. This means that alternatives to morphological characters are essential to get a complete picture of the fungal diversity and ecology at any given locality and time. Indeed, DNA-based methods have been in long use for identification purposes in mycology [1].

The locus most commonly used for identification of fungi is the ITS region of the nuclear ribosomal DNA [2,3]. This region has three subloci of different rates of evolution, ITS1: highly variable, 5.8S: very conserved, and ITS2: variable to semi-conserved [4,5]. It is flanked on both sides by comparatively conserved regions (SSU [18S] and LSU [28S], respectively) which simplifies the design of primers targeted at the ITS region. Another important aspect of the ribosomal DNA is that it is a multi-copy segment such that it amplifies readily even when the initial amount of template DNA is moderate, as is often the case with environmental samples.

A branch of mycology where sequence-based methods are in common use is ectomycorrhizal community research. Ectomycorrhizae are ecologically important root-associated symbiotic relationships between plants and fungi where the fungus obtains carbohydrates for its energy supply and the plant, among other benefits, receives nitrogen and phosphorous for its metabolism [6]. Since ectomycorrhizae normally can be observed as a hyphal cap by visual inspection of the plant roots, it is possible to sort out ectomycorrhizal root tips from soil samples and extract DNA for sequencing [2]. The sequence obtained is then typically used in a similarity search against reference databases such as GenBank [7] or UNITE [8] in the hope of finding a close match for inference of taxonomic affiliation. Given that less than 1% of all fungal species have been sequenced for the ITS region [9], the similarity search often fails to produce a match close enough for reliable species identification. As a part of the scientific publication process, such unidentified sequences are normally submitted to GenBank without a full species name. If put in the right context, these unidentified sequences may represent valuable samples of fungal diversity and ecology. Interestingly, however, unidentified sequences and their potential to contribute metadata and resolution are typically left out from phylogenetic studies [10].

**Figure 1:**
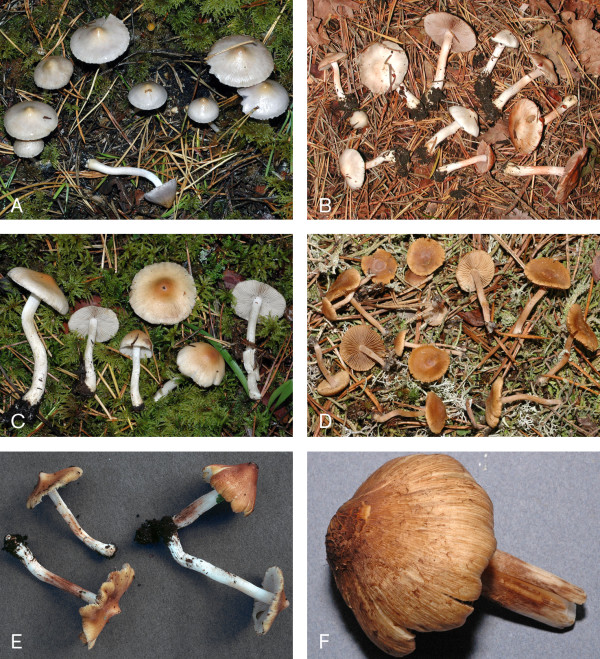
**Colour plate of *Inocybe***. A. *I. geophylla *var. *lilacina*, B. *I. whitei*, C. *I. posterula*, D. *I. jacobi*, E. *I. flavella*, and F. *I. squamata*.

One of the larger ectomycorrhizal genera is *Inocybe *(Fr.) Fr. (*Agaricales*, *Basidiomycota*; Figure 1) which comprises some 500 species [11] distributed world-wide. There are several morphology-based monographs of the genus [12,13], although most of these have a European focus (but see [14,15]). In addition, many new species have been described recently [16-20]. A search on *Inocybe *in the taxonomic database Index Fungorum [21] returns 1634 names (of species and subspecies; November 2007), but the actual number of recognized taxa is far lower owing to widespread synonymy. The fact that much of the taxonomy within the genus remains unclear [13,15,22] has been accentuated further by recent DNA-based analyses, which have shown that many of the commonly recognized taxa in the genus are incompletely understood [23,24]. In spite of the fact that *Inocybe *is a species rich, widely distributed, and commonly encountered genus, there were only 31 fully identified *Inocybe *ITS sequences deposited in GenBank at the onset of this study. The size and distribution of the genus coupled with the lack of fully identified *Inocybe *reference sequences jointly suggest that a substantial number of the unidentified ITS sequences in GenBank could belong to the genus.

This study seeks to mine GenBank for hitherto unexplored ecological and geographical data pertaining to *Inocybe*. To accomplish this, fully identified ITS sequences from an ongoing study of *Inocybe *are used together with fully identified *Inocybe *ITS sequences in GenBank to pinpoint unidentified GenBank sequences associated with the genus. The unidentified GenBank sequences are divided into groups – putative species – and identified as far as possible by sequence similarity analysis and phylogenetic inference. Information stored in GenBank about the unidentified sequences, as well as data from the papers in which they were published, are then compiled and analysed. For purposes of comparison, the number of unidentified ITS sequences associated with a selection of other ectomycorrhizal genera is estimated using fully identified GenBank sequences.

## Results

### Comparison of ectomycorrhizal genera

When comparing a selection of widespread ectomycorrhizal fungal genera it was found that *Tomentella *Pat., *Cortinarius *Fr., and *Russula *Pers. are the most commonly encountered as viewed through the number of studies that had unidentified sequences from the respective genera as their closest BLAST match (Table 1). Of these, *Cortinarius *and *Russula *are also two of the most abundantly available genera in terms of fully identified sequences in GenBank while *Tomentella *was found to be among the more poorly represented. Additionally, one of the most commonly occurring genera as unidentified sequences, *Sebacina *Tul., is among the least well represented with respect to fully identified sequences. Notably high numbers of fully identified sequences are available for *Tuber *Wigg.: Fr. and *Tricholoma *(Fr.) Quél., both of which contain species with economically important edible sporocarps (truffles and matsutake, respectively). *Pisolithus *Alb. & Schwein., the most widely used ectomycorrhizal inoculum [25], was found in only 12 studies. *Inocybe*, together with *Lactarius *Pers., are the fifth most common among the included genera in terms of the number of studies including unidentified sequences for which they form the closest match, but only the 16th most well represented with respect to the number of fully identified sequences (together with *Hygrophorus *Fr.).

**Table 1:** The estimated relative occurrence of selected ectomycorrhizal genera in GenBank based on ITS sequences

EM genus	# of studies	# of UID sequences	# of ID sequences	# of ID species
*Arcangeliella*	0	0	3	2
*Bankera*	0	0	1	1
*Phellodon*	0	0	2	2
*Sarcodon*	1	1	17	4
*Cantharellus*	1	1	22	5
*Descomyces*	1	30	7	3
*Alpova*	1	2	3	2
*Albatrellus*	3	4	35	11
*Hydnellum*	5	9	19	15
*Craterellus*	7	126	7	3
*Scleroderma*	9	14	4	3
*Xerocomus*	10	18	65	9
*Hygrophorus*	10	28	31	21
*Pisolithus*	12	68	142	7
*Tomentellopsis*	14	27	23	4
*Pseudotomentella*	16	25	12	6
*Amphinema*	18	61	4	1
*Suillus*	22	39	177	50
*Tylospora*	23	78	16	2
*Hebeloma*	25	55	117	60
*Boletus*	25	38	131	31
*Amanita*	26	51	222	84
*Cenococcum*	27	178	64	1
*Tricholoma*	29	93	286	60
*Piloderma*	30	257	44	3
*Thelephora*	31	63	20	8
*Rhizopogon*	31	316	184	51
*Tuber*	34	64	787	28
*Inocybe*^1^	42	156	31	23
*Lactarius*	48	226	272	89
*Inocybe*^2^	48	181	293	116
*Sebacina*	53	159	16	9
*Russula*	73	343	259	136
*Cortinarius*	74	521	828	398
*Tomentella*	79	463	73	26

^1 ^Before addition of fully identified *Inocybe *sequences to *emerencia *in this study.

^2 ^After addition of fully identified *Inocybe *sequences to *emerencia *in this study.

Number (#) of distinct studies with unidentified sequences associated with the respective ectomycorrhizal (EM) genera (used to order the table), the number of unidentified (UID) sequences the genera constitute the closest BLAST match for, and the number of fully identified (ID) sequences belonging to them. Based on GenBank data as of 2006-10-19.

### Alignment groups

A joint 5.8S and LSU alignment of the fully identified *Inocybe *sequences was compiled to form the basis of a division of the sequences into smaller groups. A total of 18 sequences were omitted from this alignment due to insufficient sequence length. Sixteen clades from the parsimony consensus tree inferred were selected as groups for the construction of ITS alignments. Eleven sequences fell outside these clades and were included in the ITS alignment that featured their closest BLAST match. Five sequences that did not end up with other sequences bearing the same species annotation were additionally assigned to the alignments where sequences with the same species name were present; these five sequences were therefore found in more than one alignment. This resulted in 16 alignments with 4–41 fully identified sequences. Of the 181 unidentified GenBank sequences with an *Inocybe *as the closest BLAST match, four were excluded from further analysis due to ambiguous establishment of homology in the alignment stage and the likely connection to other genera after further investigation of the BLAST output. The remaining 177 sequences were added to the alignment featuring their closest BLAST match. After the unidentified sequences and outgroup taxa had been added, the 16 alignments comprised 8–71 sequences (Additional file 1, TreeBase study accession no. S1968).

### ITS similarity

**Figure 2:**
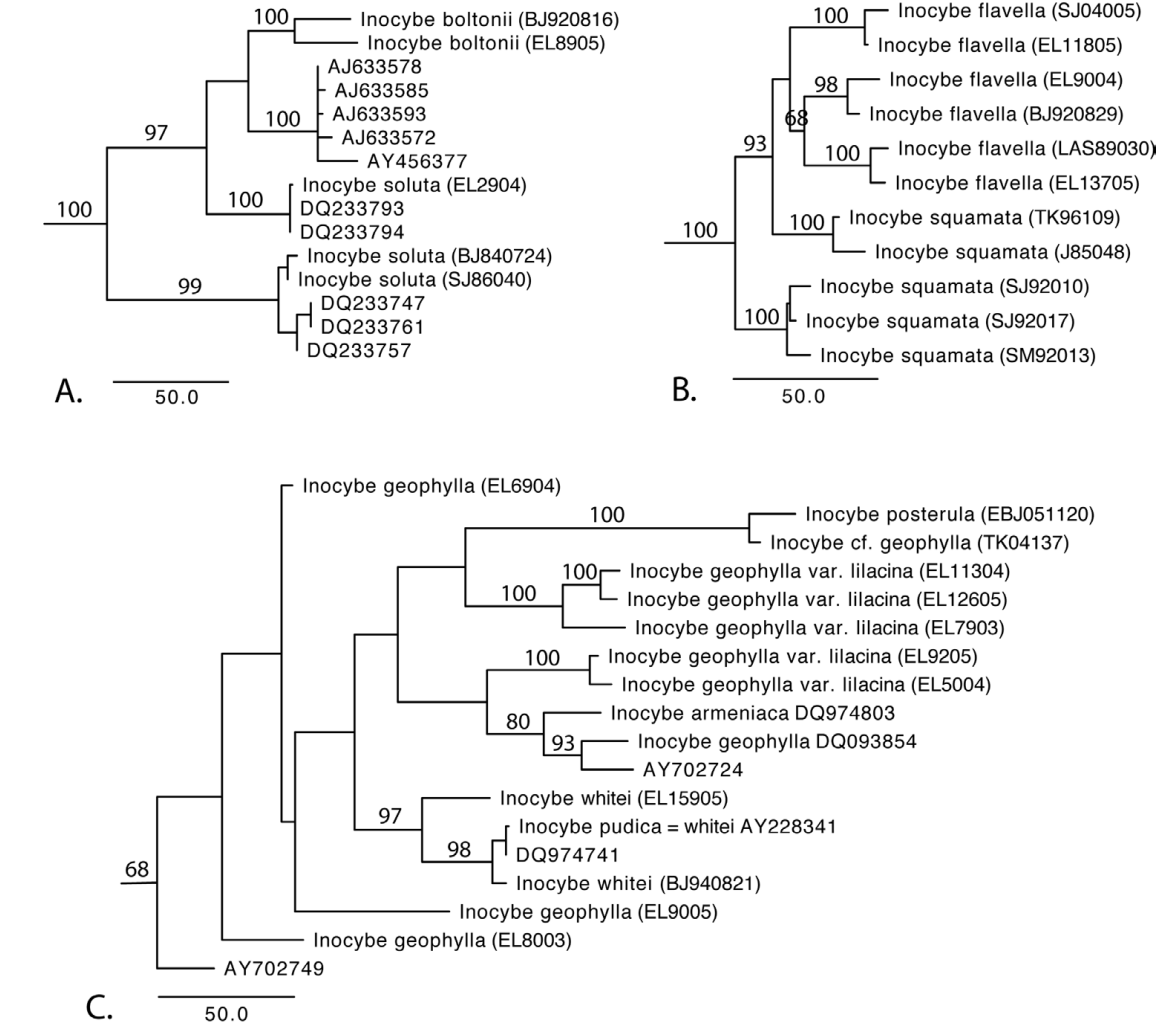
**Phylograms depicting three species complexes of *Inocybe***. The figures are derived from one of the most parsimonious trees for the respective alignment group (Additional file 1). These are based on ITS and partial LSU with jackknife support reported above the branches. When only accession numbers are given, the corresponding sequence represents an unidentified GenBank sequence; when both an accession number and a species name are given, the entry corresponds to an fully identified GenBank sequence; and when a voucher is given in parenthesis, the sequence corresponds to a sequence added in this study (Additional file 2). a) The *I. soluta*/*I. boltonii *complex, b) The *I. geophylla*, *I. posterula*, and *I. whitei *complex (in this study collectively referred to as *I. geophylla *s. l.), and c) the *I. flavella*/*I. squamata *complex.

Sixteen species of *Inocybe *(*I. acutella, I. alpigenes, I. asterospora, I. cervicolor, I. cookei, I. dulcamara, I. flocculosa, I. fraudans, I. fuscidula, I. glabripes, I. lacera*, *I. lanuginosa*, *I. maculata*, *I. muricellata, I. nitidiuscula*, and *I. rimosa*) were found to have outlier sequences that differed considerably in sequence similarity from, and were not monophyletic with, the majority of the sequences annotated as their conspecifics. These deviant sequences (but not the majority of the sequences with the same species annotation) were excluded from the analysis of intra- and interspecific similarity. Some species, such as *I. flavella, I. geophylla, I. soluta*, and *I. squamata*, were not monophyletic in the phylogenetic analysis (Figure 2) and were therefore excluded from the intra- and interspecific similarity analysis. Due to the low resolution within the clade including *I. geophylla*, *I. posterula*, and *I. whitei *(Figure 1A–C; Figure 2C), these taxa were treated together as *I. geophylla *s. l. Several species show considerable variation in the ITS2 region (<90% intraspecies sequence similarity) even within monophyletic groups. These include *I. asterospora*, *I. calamistrata*, *I. cervicolor*, *I. cookei, I. glabripes*, *I. godeyi, I. hirtella*, *I. maculata, I. mixtilis*, *I. phaeocomis*, *I. rimosa*, and *I. whitei*. For *I. calamistrata, I. cookei, I. godeyi*, and *I. maculata*, the minimum interspecific sequence similarity was smaller than the maximum intraspecific sequence similarity. The ITS1 region was found to be more variable than ITS2 in 49% of the *Inocybe *species while the ITS2 region was more variable in 31%. For 6% of the species it was not consistent which region that had the highest sequence similarity between different sequence pairs within the species. In 14% of the species both regions were fully conserved (100% sequence similarity).

**Figure 3:**
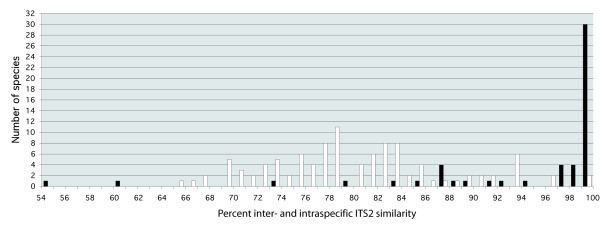
**Graph depicting inter- and intraspecific similarity for *Inocybe***. The bars represent the number of species within each interval and are based on 105 species for the interspecific similarity (white bars) and 53 species for the intraspecific similarity (black bars).

The inter- and intraspecific comparison (Figure 3) of sequence similarity in ITS2 indicates that a cut-off value of about 97% would give the most reasonable error rates for species delimitation in *Inocybe*: 71% of the species had an intraspecific sequence similarity larger than 97%, and 98% of the species had an interspecific similarity lower than 97%. This fits well with previously used cut-off values in mycology [26]. There were two cases where unidentified sequences from the same study formed clades with full jackknife support but without internal resolution in the phylogenetic analysis and where the sequence similarities were between 96% and 97%. Since it seems likely that these belong to the same species and the effect on the error estimates would be small, a 96% cut-off value was used instead of 97%. This had no effect on the identity of the remaining unidentified sequences.

The ITS1 region indicated the same sequence groups as ITS2 except for one of the unidentified sequences [GenBank:AY970244] that showed affinities to different sequence groups for the two different regions.

### Species identification

Of the 177 unidentified GenBank sequences with an association to *Inocybe*, 56 (32%) were identified to species level in this study, 21 (12%) were identified as having a close affinity to a fully identified *Inocybe *species (corresponding to the denotation *aff*.), and 100 sequences (56%) remains unidentified (annotated as *sp*. or *cf*.; Additional file 2). The 31 fully identified *Inocybe *sequences originally in GenBank constitute 16 (9%) of the closest BLAST matches after the addition of the new *Inocybe *sequences. The unidentified sequences of *Inocybe *were determined to belong to 98 different species. The study with the largest number of *Inocybe *species, as circumscribed from unidentified sequences here, featured 13 species and was conducted in a meadow ecosystem on calcareous soil in western Estonia [27].

Ninety-four (53%) of the 177 unidentified GenBank sequences assigned to *Inocybe *in this study were from a published study that assigned an identity to the sequence. Of these 94 sequences, eight (9%) were determined to species level in the study they were presented, 49 (52%) were identified as *Inocybe*, 12 (13%) as *Cortinariaceae*, and 22 (23%) at an even higher taxonomic level (or as completely unidentified). There were also three sequences (3%) that had been classified to taxonomic ranks that do not include *Inocybe*.

**Figure 4:**
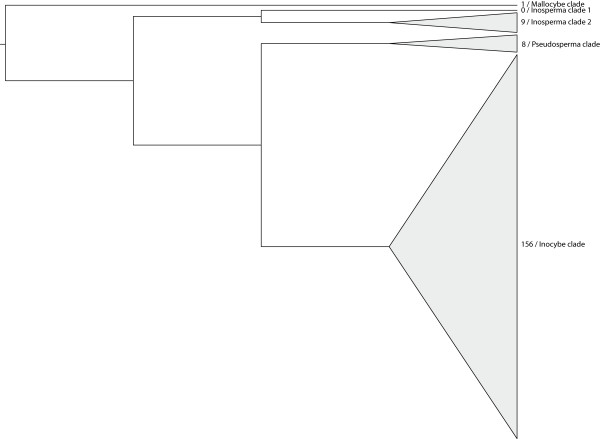
**Schematic illustration of the distribution of unidentified sequences among the major clades of *Inocybe***. The clades are named according to [23] and the numbers depict the number of unidentified sequences associated with each clade. As no fully identified ITS sequences representing species in the *Auritella *clade were available, this clade was excluded.

There was a marked skewness in the distribution of unidentified ITS sequences among the *Inocybe *clades given in [23] (Figure 4), with the vast majority (156/177) of the sequences belonging to the *Inocybe *clade, only one to the *Mallocybe *clade (subgenus *Mallocybe *Kuyp.), and none to the *Inosperma *clade 1 (section *Cervicolores*). Two sequences were found to have potential affinities to the *Inosperma *clade and one to the *Pseudosperma *clade but could not be confidently included in these and were therefore excluded from the analysis.

### Geography and ecology

**Figure 5:**
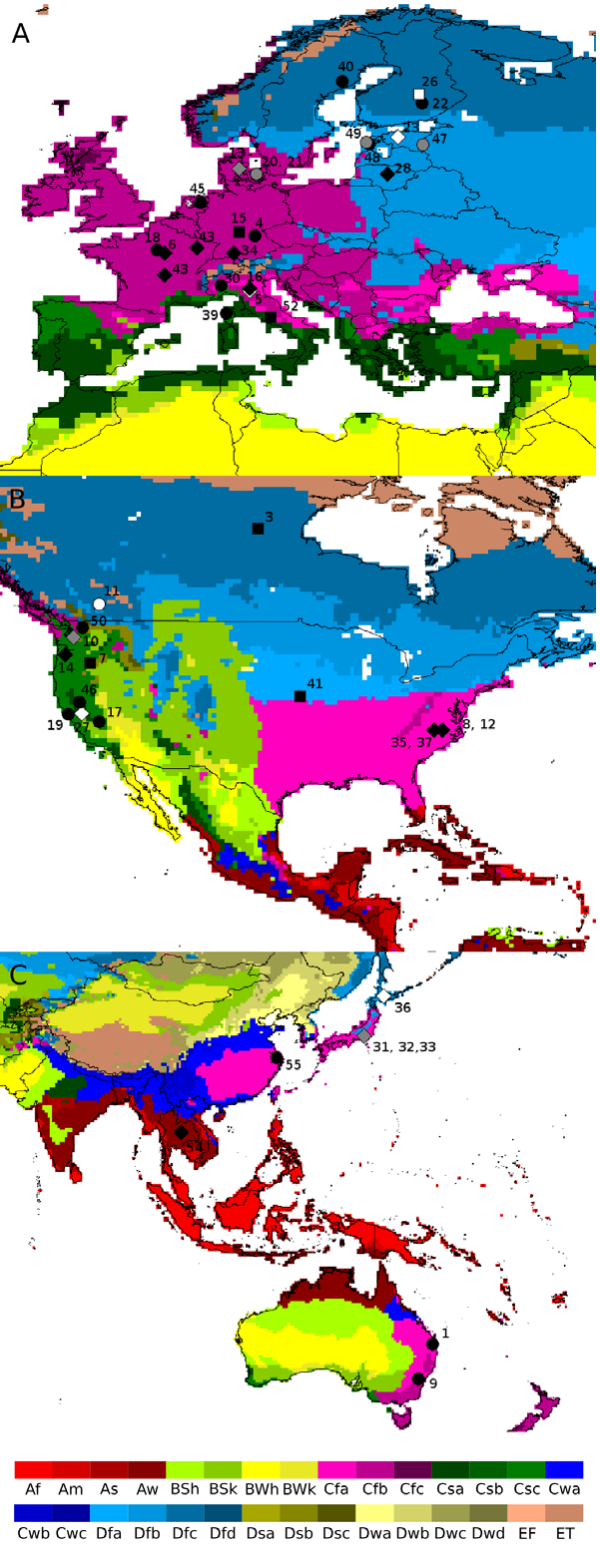
**The geographic origins of *Inocybe *sequences (present in GenBank) that could be geographically assessed**. a) North America, b) Europe, c) Asia and Australia. The color coding of the map represents climate according to the Köppen-Geiger climate classification (modified from [28]); main climate A = equatorial, B = arid, C = warm temperate, D = snow, E = polar; precipitation W = desert, S = steppe, f = fully humid, s = summer dry w = winter dry, m = monsoonal; temperature h = hot arid, k = cold arid, a = hot summer, b = warm summer, c = cool summer, d = extremely continental, F = polar frost, T = polar tundra. Black symbols represent studies with unidentified sequences, white symbols represent studies with fully identified sequences, and grey symbols represent studies with both unidentified and fully identified sequences. Circles represent studies for which the coordinates were given in the paper in which they were published, diamonds represent studies where the location had to be inferred from the locality given in the paper, and squares represent studies where the location was not available beyond the country/state level. The numbers correspond to the number given to each study in the Additional file 2.

The majority of studies in which *Inocybe *has been reported was conducted in Europe and North America (Figure 5). Four studies could not be confidently geographically assigned to a continent and were therefore excluded from all analyses regarding geography and climate, and an additional five studies could not be confidently assigned to a climatic region and were therefore excluded from all climatological analysis. A disproportionately large number of the unidentified sequences that were successfully identified in this study came from Europe: 49% of the European sequences were identified to species level whereas for N. America the corresponding number was 25%. For Asia and Australia the values were 14% and 0% respectively, but both areas were represented by fewer than ten sequences. When comparing the Köppen-Geiger climate regions [28] that were represented by more than one study containing *Inocybe *sequences, 50% of the unidentified sequences in fully humid snow climate (cold temperate; Df) were identified to species level here; for fully humid warm temperate climate (Cf), 30% were identified; and for warm temperate climate with dry summers (Mediterranean climate; Cs), 32% were identified (Table 2; Figure 5).

**Table 2:** The number of *Inocybe *species/sequences on the different continents and in the different climatic regions

		N. Am.		Asia		Aus.		Eur.		Total	
		Spec.	Seq.	Spec.	Seq.	Spec.	Seq.	Spec.	Seq.	Spec.	Seq.

Df	#			2	2			18	28	20	30
	%			0	0			39	54	35	50
Ds	#	1	1							1	1
	%	0	0							0	0
Cf	#	11	32	2	2	4	8	21	28	37	70
	%	27	9	50	50	0	0	52	61	38	30
Cs	#	27	38					3	3	30	41
	%	19	34					0	0	17	32
Aw	#			3	3					3	3
	%			0	0					0	0
?	#	4	6					8	9	12	15
	%	50	50					13	11	25	27
Total	#	46	77	7	7	4	8	46	68		
	%	28	25	14	14	0	0	37	49		

Df = snow climate, fully humid; Ds = snow climate with dry summer; Cf = warm temperate climate, fully humid; Cs = warm temperate with dry summer; Aw = equatorial savannah with dry winter; ? = climatic region uncertain. N. Am. = North America; Aus. = Australia; Eur. = Europe. Spec. = species; Seq. = sequences. # = number of unidentified *Inocybe *species/sequences; % = percentage being identified in this study.

Only 19 (19%) of the species delimited from unidentified GenBank sequences were found in two or more studies. The species found most often was *I. glabripes *which occurred in five studies, although two of those were conducted in the same forest. Five species (*I. glabripes*, *I. lanuginosa*, *I. maculata*, *I. rimosa*, and *I. sp*. 31 [Additional file 2]) were found in both Europe and North America (six if counting one species [*I. jacobi*, Figure 1D] whose location was inferred from the title of the study given in GenBank [Additional file 2]) and one species was found both in Europe and Japan (I. sp. 2 [Additional file 2]). In addition, eight (nine, as previous) of the species that were identified to species level in this study were collected outside Europe (where at least one of the fully identified sequences they group with originated from).

A total of 51 species were found in hardwood forests, 24 in coniferous forests, and 14 in mixed forests. Two of these species were found in both hardwood and coniferous forests, two in both hardwood and mixed forests, and five in both coniferous and mixed forests. The host was given either in GenBank or in the paper from which the sequence originated for 70 (40%) of the sequences representing 52 species. Eight of those species were found on more than one host genus. Among the more noteworthy hosts are *Arbutus unedo *and *Orthilia secunda *(*Ericaceae*), which form arbutoid mycorrhizae, and species of *Cephalanthera *and *Epipactis *(*Orchidaceae*), which exhibit orchid mycorrhizae [6,29]. Oddly, one *Inocybe *sequence comes from a study of the total mycoflora of the grass *Phragmites australis *(*Poaceae*) [30]. The rest of the hosts were known ectomycorrhizae formers (Additional file 2). Three species were reported from ecological conditions differing from those in the literature: *I. lanuginosa*, *I. leptophylla*, and *I. phaeocomis (*Additional file 2).

## Discussion

*Inocybe *is one of the larger genera of ectomycorrhizal fungi and its species are consequently found in many studies of ectomycorrhizal communities. The comparison between the genera in this study is based on the closest BLAST matches for the unidentified sequences, an approach that although associated with complications [10] serves as a crude quantification of the frequencies at which the genera occur. The number of fully identified ITS sequences annotated as *Inocybe *in GenBank was similar to those of the likewise ectomycorrhizal genera *Hygrophorus *and *Albatrellus *Gray that form the closest BLAST matches of unidentified sequences from much fewer studies. As many as 58% of the sequences found to be associated with *Inocybe *were not denoted as such in the study in which they were originally published, which indicates that the lack of reference sequences of *Inocybe *has severely impeded identification to genus level.

The observation that seven (37%) of the 19 species recovered in more than one study were found on different continents suggests that at least many of the north temperate *Inocybe *species are widely distributed. This finding notwithstanding, it is clear that the restricted geographical area from which the fully identified sequences were collected has had an impact on the number of unidentified sequences being identified to species level. In this study, fully identified sequences belonging to 116 different *Inocybe *species were included, a set that represents about 20% of the estimated number of described species in the genus. If this was a representative taxonomic sample for the world it should be a reasonable basis for species identification, but when comparing different geographical regions it was only in fully humid temperate and snowy (cold temperate) parts of Europe that a significant proportion of the unidentified sequences was successfully identified in this study. With respect to sequences from Australia and Thailand, none of the unidentified sequences were identified to species level in this study, indicating a geographic skewness in the taxonomic sampling.

Since there is a general bias in the geographic distribution of ectomycorrhizal studies towards Europe and North America [31], it is hard to draw precise conclusions on the distribution of *Inocybe*. It is nevertheless clear from the results that the genus is widely distributed and can be found in many climatic regions, from fully humid snow (cold temperate) climate to equatorial savannah. It furthermore appears that *Inocybe *can form mycorrhizal associations with various diverse hosts and not only of the ectomycorrhizal type but also arbutoid and orchid mycorrhizae [6]. In addition, many species were found to form mycorrhizae with more than one host genus, which expands on what has previously been reported for *Inocybe *[12]. The study in which an *Inocybe *sequence was recovered from a tissue sample of the grass *Phragmites australis *[30], a species that forms arbuscular mycorrhizae [29], was designed to investigate general fungal associates with the plant and not specifically mycorrhizal associations. There are previous reports that *I. serotina *can be associated with *Ammophila arenaria *[12], another arbuscular mycorrhizal member of *Poacea *[29]. Since arbuscular mycorrhizae is restricted to the phylum *Glomeromycota *among the fungi [6], it seems unlikely that the association between *Inocybe *(*Basidiomycota*) and these grasses is mycorrhizal. This might suggest that the *Inocybe *species in question may have colonized the host for other reasons (or were accidentally amplified from the surrounding matrix) such that not all species of *Inocybe *are obligately mycorrhizal.

Most of the unidentified ITS sequences with an affiliation to *Inocybe *were found to be associated with the *Inocybe *clade (subgenus *Inocybe*; Figure 4). This is not surprising since this is the largest clade within the genus in terms of the number of species [13]. The observation that only one sequence was associated with the *Mallocybe *clade (subgenus *Mallocybe*) and none with the *Inosperma *clade 1 (section *Cervicolores*; Figure 4) might be due to a bias in what kind of ecosystems that have been sampled combined with random sampling effects, but it could also be interpreted as an indication of a different nutritional mode for the species of these clades.

Species identification using DNA sequences from only one locus has been cautioned against for several reasons [32,33]. In this study we grouped sequences based on a dual demand of sufficient sequence similarity and monophyly in a phylogenetic context. Such groups might be more correctly referred to as molecular operational taxonomic units (MOTUs [34]) and it remains to be shown how well they correspond to actual species and whether discrepancies between these units will have an impact on our results. If a species is represented by only one or a few sequences (and that is often the case), species determination and delimitation may have to rely on similarity threshold values, a process that while known not always to be reliable [35,36] has been shown to be useful in some groups of fungi [37,38]. In this study, threshold values were inferred from the sequence similarity among the fully identified sequences, but the variation in inter- and intraspecific similarity among the species was found to be large and at times overlapping, resulting in similarity thresholds that are a compromise between over- and underestimating the number of species. Some of this variation in sequence similarity might be explained by insufficient taxonomic resolution such that one species name is applied to what is actually several species, consequently with an overestimation of the intraspecific variability as a result.

In *I. rimosa *and *I. maculata*, the low sequence similarity is accompanied by a large variability in morphology and uncertain taxonomy. *Inocybe rimosa *is known to be a taxonomically difficult species with different opinions on species delimitation [12]. This study also reveals that there is large ecological variation, including associations with several hosts, among the collections of *I. rimosa*, which emphasizes the need for more data to settle the taxonomy within this complex. For *I. maculata*, Kuyper [12] reports a great variation in colour, and there are also some species with purportedly close affinities to *I. maculata *that have not been considered in this study (e.g., *I. fastigiella *and *I. lanatodisca*). The three species *I. phaecomis*, *I. hirtella*, and *I. cookei *are each divided into varietal taxa reflecting a marked morphological diversity that has only partly been accounted for in the present study. For other species, the variation in sequences is harder to correlate to morphological diversity.

The multiple copies of the nuclear ribosomal DNA undergo concerted evolution that serves to homogenize the array of copies. Sequence similarity measures based on the ITS region are complicated by the fact that there may still be several distinct and mutually heterogeneous copies of the region in one genotype [39], adding to the sequence variation observed within a taxon. Another possible source of variation in sequence similarity is sequencing errors [40], but since primary data (raw sequences) rarely are easily available in the public databases, it is hard to judge the quality of a sequence and thus the extent of this problem. In this study we found one sequence that associated with different sequence groups depending on which of the ITS subregions that were considered. This could be explained if the sequence represents an in situ hybridization between two ITS types or if the sequence is chimeric [39].

Another pitfall in single-locus sequence-based species identification is that species do not always appear to form monophyletic groups in phylogenetic analyses [41] (and do not always have the highest sequence similarity with conspecific sequences). There might be several explanations for this, such as poorly resolved phylogenetic estimates, inadequate taxonomy, and incomplete lineage sorting [42,43]. There were several cases in the present study where sequences with identical species annotation were found not to form monophyletic groups, and some sequences fell well inside the clades of other species. Most of these cases are probably due to misidentification, and in a few instances it is plausible that the same name is used for different species in different parts of the world [44]. This points to the importance of voucher specimens so that such disagreements can be emended [45]. There are some instances, however, where other explanations are conceivable. *Inocybe boltonii *and *I. aff. boltonii *render *I. soluta *paraphyletic and divided into two clades. The nodes between the clades within this complex are well supported and additional information from morphological studies or other loci is needed to resolve the reason for this non-monophyly. Specimens annotated as *I. flavella *and *I. squamata *(Figure 1E,F) form a clade where each species is divided into more than one separate clade. Although the phylogenetic relationships are not fully resolved for these taxa, it is clear that at least *I. squamata *is non-monophyletic. This could be explained by incomplete lineage sorting, hybridisation/introgression, or inadequate taxonomic resolution [41,46]. Another complex of species, referred to as *I. geophylla *s. l. here, was circumscribed to include several taxa. Two of these taxa do not form monophyletic groups, but the resolution within this complex of species is poor. The low sequence similarities among the different clades implore further taxonomic studies.

## Conclusion

As more and more sequences are made available from environmental samples, this new and previously unexplored data can be used by systematists as an information resource on the ecology and distribution of different taxa. These sequences also represent a valuable sample of intraspecific diversity and might furthermore present clues on where to look for previously undescribed species, but in the end it is the standard and level of detail of the metadata for each sequence that will determine the ease and extent to which this information can be utilized. As this study shows, it is vital to provide rich and consistent sequence metadata even if the sequences are not identified to species level, and much would be gained if all sequence entries in GenBank were thus annotated.

## Methods

A number of sequences from fruiting-bodies identified on morphological basis were made available for use in this study from an ongoing phylogenetic investigation of *Inocybe *in northern Europe (Larsson *et al*., unpublished). The regions used were the ITS and LSU of the nuclear ribosomal DNA [4]. A set of 294 sequences of *Inocybe *was selected from this study to represent 109 species and all subgenera sensu [12,13] and all the major clades from [23] except the *Auritella *clade (Additional file 2). Two outgroup taxa were chosen from the closely related genera *Crepidotus *(Fr.) Kumm. and *Naucoria *(Fr.) Kumm. [24,47]. Names are given according to Kuyper [12] and Stangl [13] as far as applicable; *I. obsoleta *and *I. perlata *are, however, recognized as separate species.

### Integration in emerencia

A local installation of *emerencia *1.0 [9] was used to download all fungal ITS sequences from GenBank and to divide these into fully identified (45 909 sequences, of which 31 were annotated as *Inocybe*) and unidentified (21 738 sequences) with respect to species level annotations. The sequences were stored in a MySQL 4.1.11 database. Of the 294 sequences added in this study, 262 (109 species) were included in the table for fully identified sequences and 32, denoted as *cf*. or *sp*., were included in the table for unidentified sequences (Additional file 2).

Since dubiously annotated sequences constitute a challenge for users of GenBank [48], all fully identified sequences were compared for similarity against each other using BLAST 2.2.9 [49] not to miss any *Inocybe *sequences bearing other generic names. The five closest BLAST matches of all fully identified sequences were scrutinized to determine whether any of the newly added *Inocybe *sequences were among them. *emerencia *was then evoked to BLAST all unidentified sequences against all fully identified sequences. The table for unidentified sequences was queried for sequences that had a fully identified sequence belonging to *Inocybe *(including sequences annotated as *Mallocybe*) as their closest BLAST match. For purposes of comparison, this was done both before and after the addition of the *Inocybe *sequences released through this study and also for several other common mycorrhizal genera using fully identified sequences from GenBank.

### Phylogenetic analysis

To facilitate species identification of the unidentified sequences, evolutionary relationships among the sequences were investigated using phylogenetic analysis [50]. Since ITS cannot be satisfactory aligned over all *Inocybe *species, smaller groups had to be constructed to enable estimation of phylogenies based on this region. To compile suitable alignment groups, the *Inocybe *sequences classified as fully identified (according to the criteria in *emerencia*) were aligned using Clustal W 1.83 [51]. The ITS1 and ITS2 regions were excluded such that only the 5.8S (156 bp.) and the 5' end of LSU (1362 bp.) were included. Sequences with less than 200 bp. of data were excluded. PAUP* 4.0b10 [52] was employed for the parsimony phylogenetic analyses using 25 000 random addition repeats holding one tree per replicate and TBR branch swapping with at most five trees saved per replicate. The resulting trees were summarized in a consensus tree to find clades of sequences consistent with all most parsimonious trees. Based on these clades and considering the major clades of [23], groups consisting of a maximum of 50 sequences were selected as basis for the compilation of ITS alignments. Sequences falling outside these groups, as well as sequences excluded due to lack of sufficient sequence data in the 5.8S and LSU regions, were placed in the group where they found their closest BLAST match. If they were found to have conspecific sequences (as determined through their species annotation) in another group, they were included in that alignment as well. The groups were then aligned in Clustal W and adjusted manually in SeaView 1.0 [53].

The unidentified ITS sequences that found an *Inocybe *sequence as their closest BLAST match were aligned to the ITS alignment that featured that matching sequence using Clustal W. *Naucoria submelinoides*, *Crepidotus mollis*, and *I. dulcamara *were used as outgroups for all but one alignment corresponding to the subgenus *Mallocybe *[12] in which *I. dulcamara *was included in the ingroup [23]. The alignments were analysed under the parsimony criterion in PAUP* using 1 000 random addition repeats holding two trees per replicate and TBR branch swapping with at most 30 trees saved per replicate. For alignments with fewer than 22 sequences, the exact branch and bound search option was used instead. The MAXTREES option was set to 30 000. The resulting trees were summarized in a strict consensus tree. Branch support was estimated through 1 000 jackknife replicates with the JAC resample method, independently deleting 37% of the characters in each step [54]; the heuristic search settings for the jackknife analysis were set to 100 random addition replicates holding one tree per replicate and TBR branch swapping with at most five trees saved per replicate. For alignments with fewer than 20 sequences, branch and bound was used. Alignments and trees were deposited in TreeBASE.

### Sequence similarity

Sequence similarities (expressed as Hamming distances [55]) were obtained from pairwise alignments generated by Clustal W. Leading and trailing gapped parts of the alignments were excluded from the comparison, and the similarities were calculated in Perl.

All fully identified *Inocybe *sequences and unidentified sequences with an *Inocybe *sequence as their closest BLAST match were compared for similarity in ITS2, and similar sequences were then grouped together using single link clustering [56] using several different cut-off values. To estimate the error of false lumping of species for different cut-off values, the minimum interspecific pairwise sequence similarity in ITS2 was computed for all fully identified species. Likewise the maximum intraspecific similarities were computed for all fully identified species represented by more than one sequence to estimate the error of false splitting of species. Sequences not forming monophyletic groups with respect to the species annotation were omitted from these error estimations.

The sequence similarity for the ITS1 and ITS2 regions was computed among the sequences in each of the ITS alignments. The patterns of similarity in ITS1 were then compared with those of ITS2. Sequences with less than 100 bp. in any of the investigated regions were omitted from all the similarity analyses.

### Taxonomic affiliation of the sequences

The taxonomic affiliation of the unidentified sequences was inferred considering both the phylogenetic trees and the similarity clusters. If an unidentified sequence was assigned to a clade of homogeneously named, fully identified sequences with significant jackknife support (≥70%), it was assigned to that species. When comparing the different cluster analyses, the estimated error rates were used to choose what cut-off value to apply. If a sequence appeared in a cluster with homogeneously named, fully identified sequences, it was assigned to that species if this was not contradicted by the phylogenetic analyses. Similarly to the analysis of intra- and interspecific sequence similarity, all fully identified sequences not forming monophyletic groups with respect to their species annotation were excluded when inferring taxonomic affiliation of the unidentified sequences. Unidentified sequences appearing in the same cluster were considered to be the same species if this was not contradicted by the phylogenetic analysis. If an unidentified sequence appeared as sister taxon to a fully identified species in the phylogenetic analysis and had at least 80% sequence similarity in ITS2, it was denoted as *aff*. of that species (e.g. *I. aff. boltonii*). Unidentified sequences that were deemed to belong to the same species (according to the criteria above) as partly identified sequences annotated as *Inocybe cf*. and a species epithet were also annotated as *I. cf*. and that species epithet (e.g. *I. cf. fuscidula*). Sequences annotated as *cf*. or *aff*. were treated as unidentified.

### Ecology and geography

Information about the ecology of the unidentified sequences was compiled from the papers in which they were published and from the respective annotations in GenBank. To determine in what climatic region each study was conducted, the locations of the studies that could be geographically assessed were plotted on a map of the Köppen-Geiger climate classification [28]. This information was then compared with [12,13,57].

## Abbreviations

ITS – Internal transcribed spacer; LSU – Large subunit; SSU – Small subunit; BLAST – Basic local alignment search tool; bp. – base pair(s).

## Authors' contributions

MR had the main responsibility for the project and participated in all parts of it. RHN was responsible for setting up the local installation of *emerencia*, assisted with database handling, and participated in the phylogenetic analysis. EK was responsible for the statistics and conducted the cluster analysis. MT was responsible for GIS applications and constructed the maps. SJ and EL had the main responsibility for taxonomic issues and EL did most of the molecular work. All authors drafted the manuscript and approved the final version.

## Supplementary Material

Additional file 1 **Trees depicting each of the 16 alignment groups**. Phylograms representing one of the most parsimonious trees (including jackknife support values) for each of the 16 alignment groups. Click here for file

Additional file 2 **Metadata associated with *Inocybe *sequences in GenBank**. Unidentified (Table A) and fully identified (Table B) *Inocybe *sequences from GenBank included in this study; the studies they originate from (Table C); the ecology, as given in Kuyper [12], Stangl [13], and Hallingbäck and Aronsson [57], of the species they were determined to belong to (Table D); and sequences added in this study (Table E). Click here for file
