# Supplementary material for: Mining metadata from unidentified ITS sequences in GenBank: A case study in Inocybe (Basidiomycota)
Source: BMC Evol Biol. 2008 Feb 18;8:50. doi: 10.1186/1471-2148-8-50 (PMC2275786; doi:10.1186/1471-2148-8-50)
Supplement: Additional file 1 — Trees depicting each of the 16 alignment groups. Phylograms representing one of the most parsimonious trees (including jackknife support values) for each of the 16 alignment groups. [file 1471-2148-8-50-S1.pdf]

Additional tree figures

Phylograms depicting the 16 alignment groups of Inocybe used in this paper. The tree figures represent one of the most parsimonious trees for the respective alignment group. These are based on ITS and partial LSU. Jackknife support values above 50 are reported above the branches but have been omitted on some very short branches in the interest of readability. When only accession numbers are given, the corresponding sequences represent unidentified GenBank sequences; when both accession number and species name are given the entry corresponds to an identified GenBank sequence; and when a voucher is given in parenthesis the sequence corresponds to a sequence added in this study.

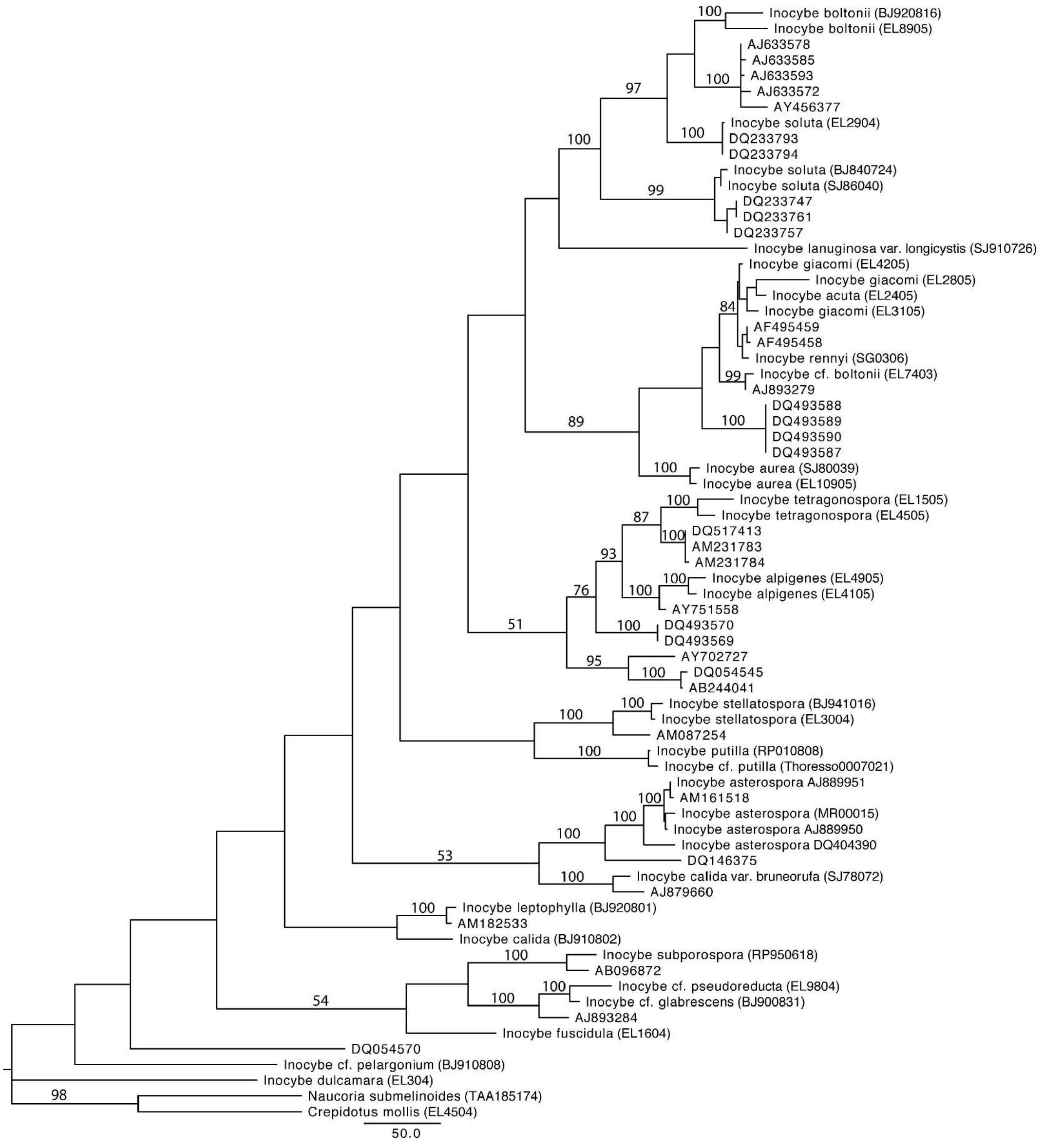

A. Alignment group 1. Sequences from Inocybe subgenus Inocybe. One of 2970 most parsimonious trees. Alignment with 2383 characters of which 1491 were constant, 250 variable but parsimony-uninformative, and 642 were parsimony-informative. The tree is 3002 steps long, CI = 0.5080, RI = 0.6970.

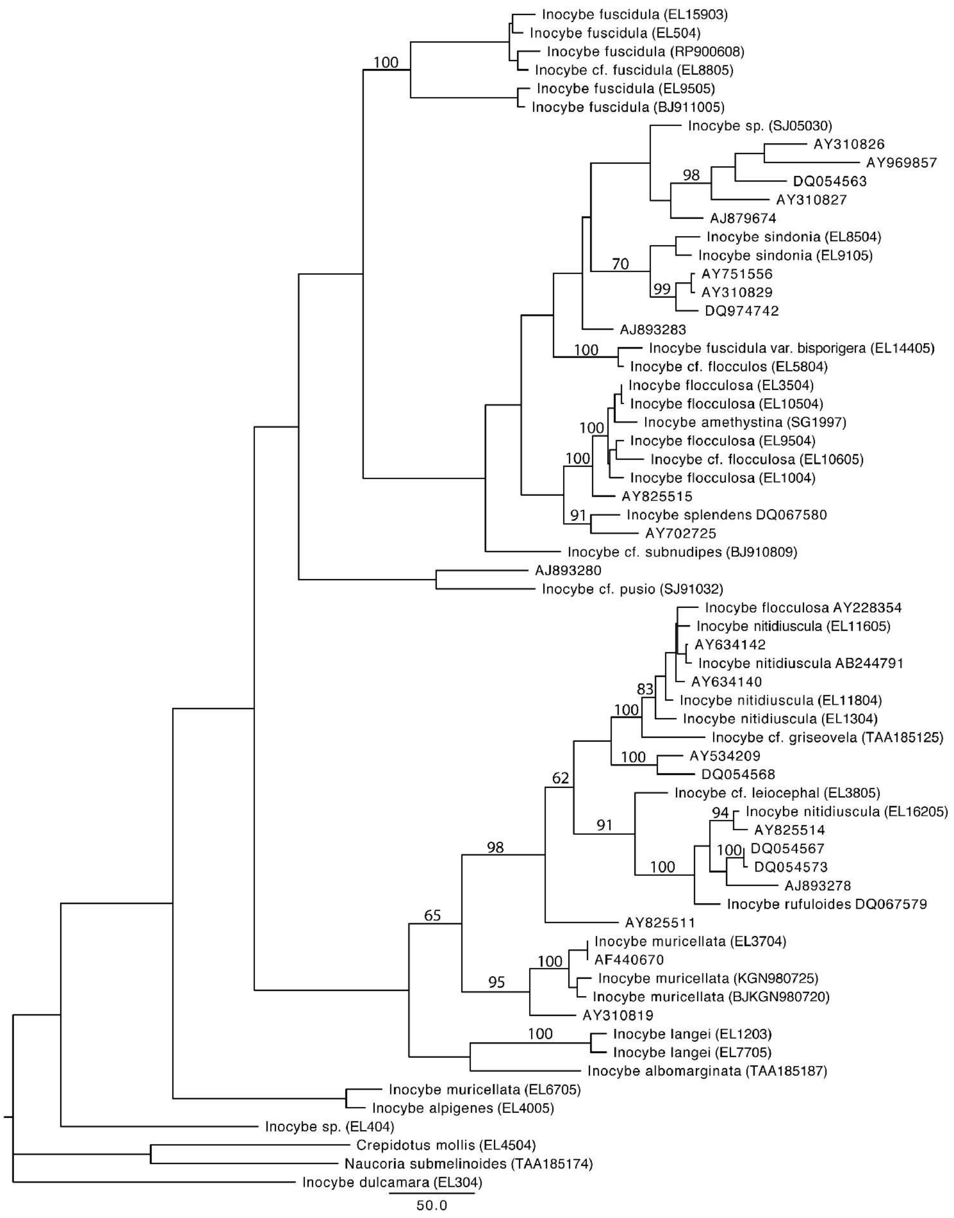

B. Alignment group 2. Sequences from Inocybe subgenus Inocybe. One of 6066 most parsimonious trees. Alignment with 2276 characters of which 1431 were constant, 291 variable but parsimony-uninformative, and 554 were parsimony-informative. The tree is 2785 steps long. CI = 0.4901, RI = 0.6695.

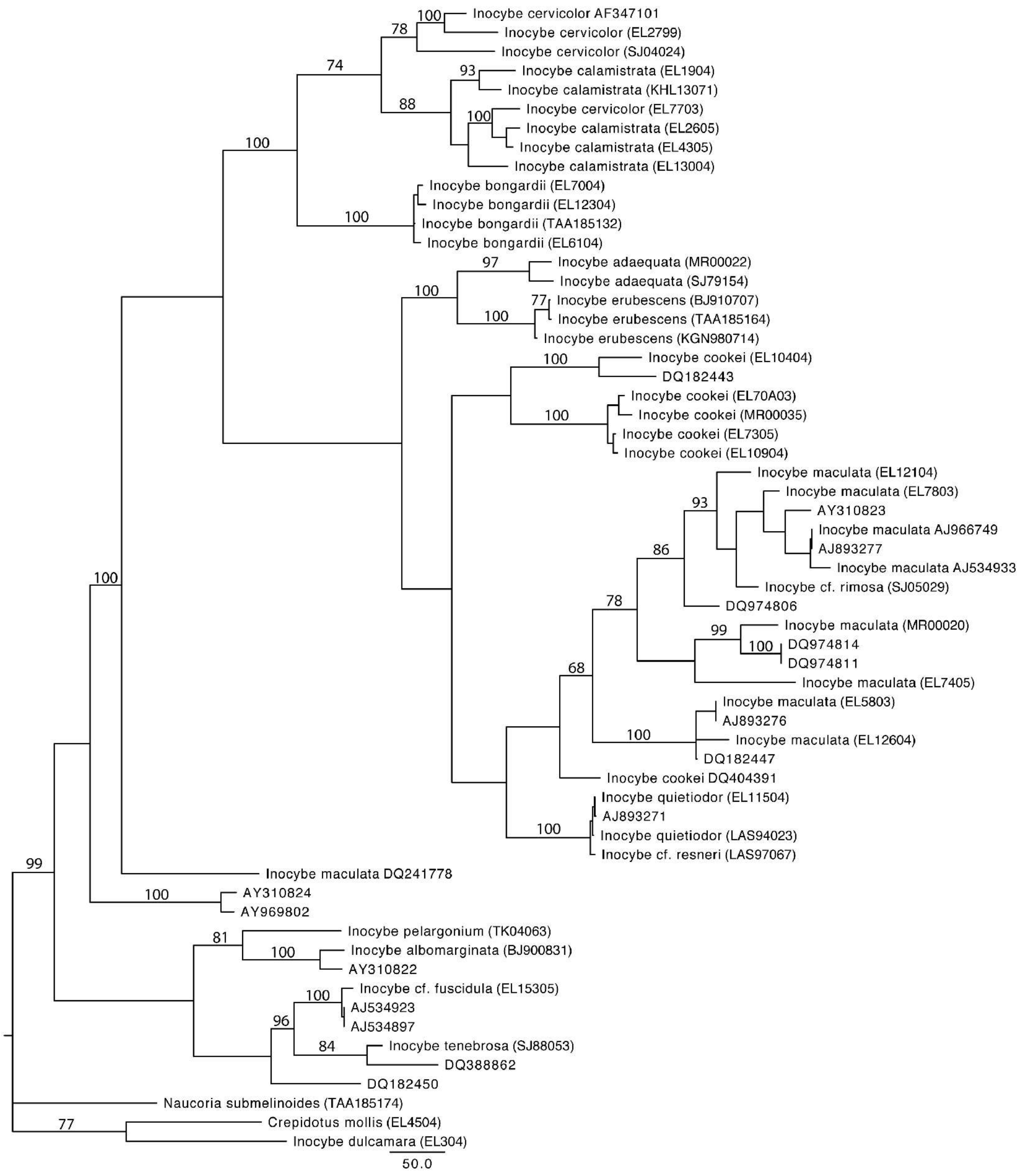

C. Alignment group 3. Sequences from Inocybe subgenus Inosperma s. s. and some sequences from subgenus Inocybe. One of 1380 most parsimonious trees. Alignment with 2282 characters of which 1310 were constant, 243 variable but parsimony-uninformative, and 729 were parsimony-informative. The tree is 3846 steps long. CI = 0.4615, RI = 0.6852.

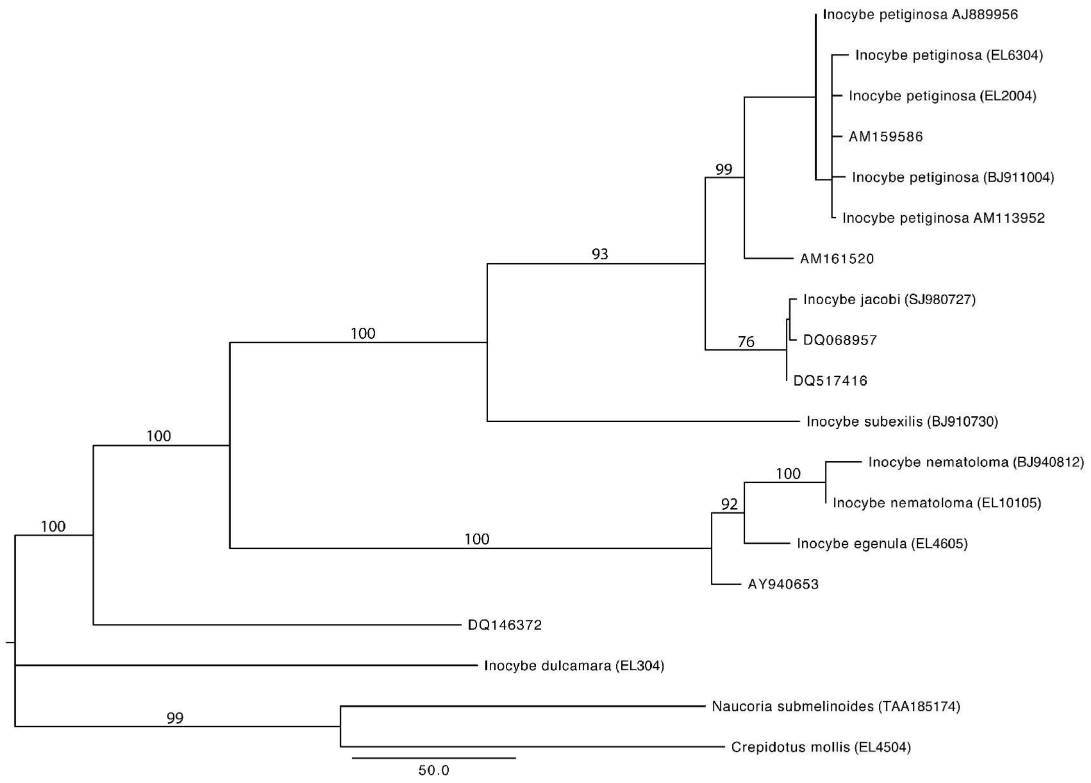

D. Alignment group 4. Sequences from Inocybe subgenus Inocybe. One of 29700 most parsimonious trees. Alignment with 2188 characters of which 1601 were constant, 299 variable but parsimony-uninformative, and 288 were parsimony-informative. The tree is 946 steps long. CI = 0.8372, RI = 0.6551.

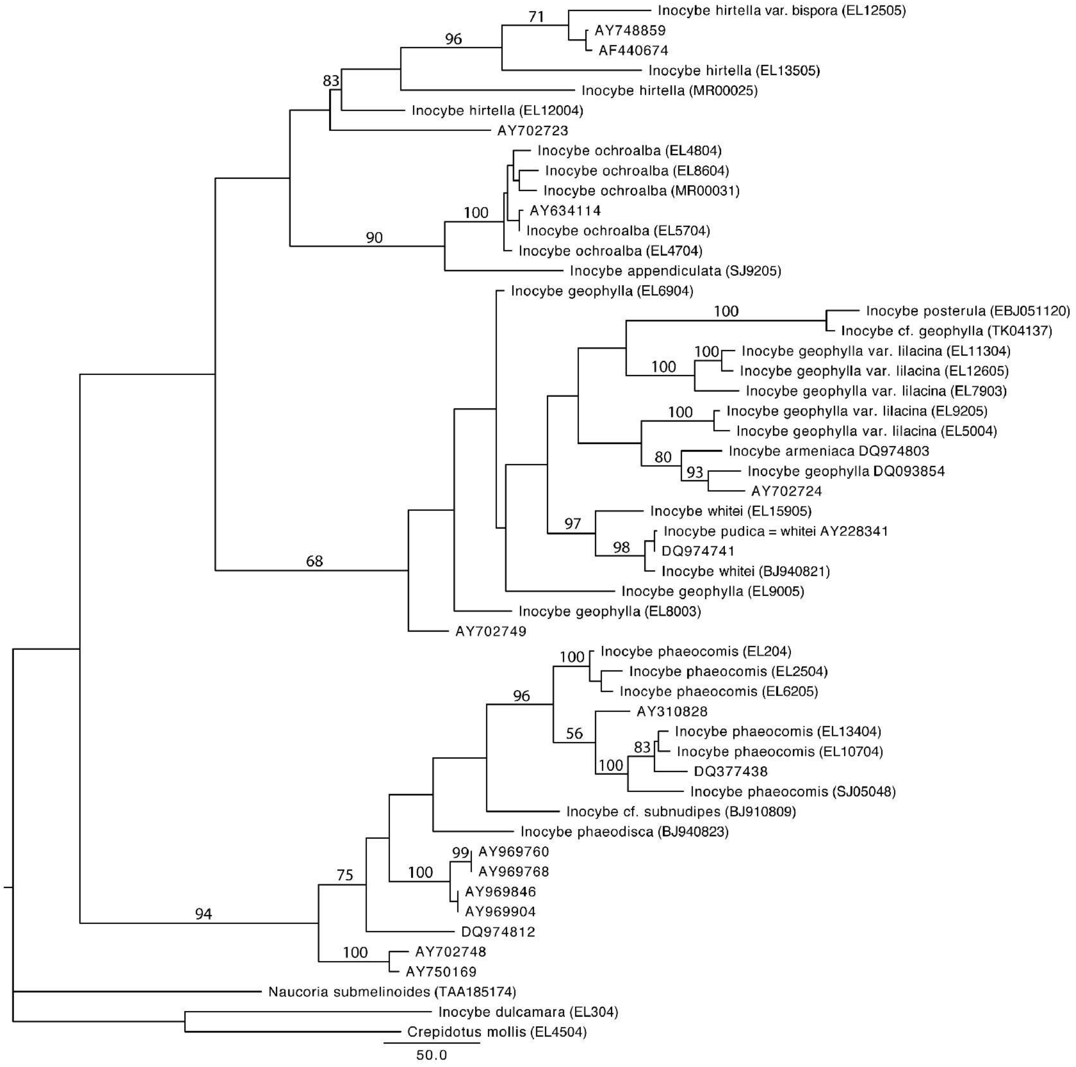

E. Alignment group 5. Sequences from Inocybe subgenus Inocybe. One of 613 most parsimonious trees. Alignment with 2289 characters of which 1472 were constant, 271 variable but parsimony-uninformative, and 546 were parsimony-informative. The tree is 2414 steps long. CI = 0.4699, RI = 0.6935.

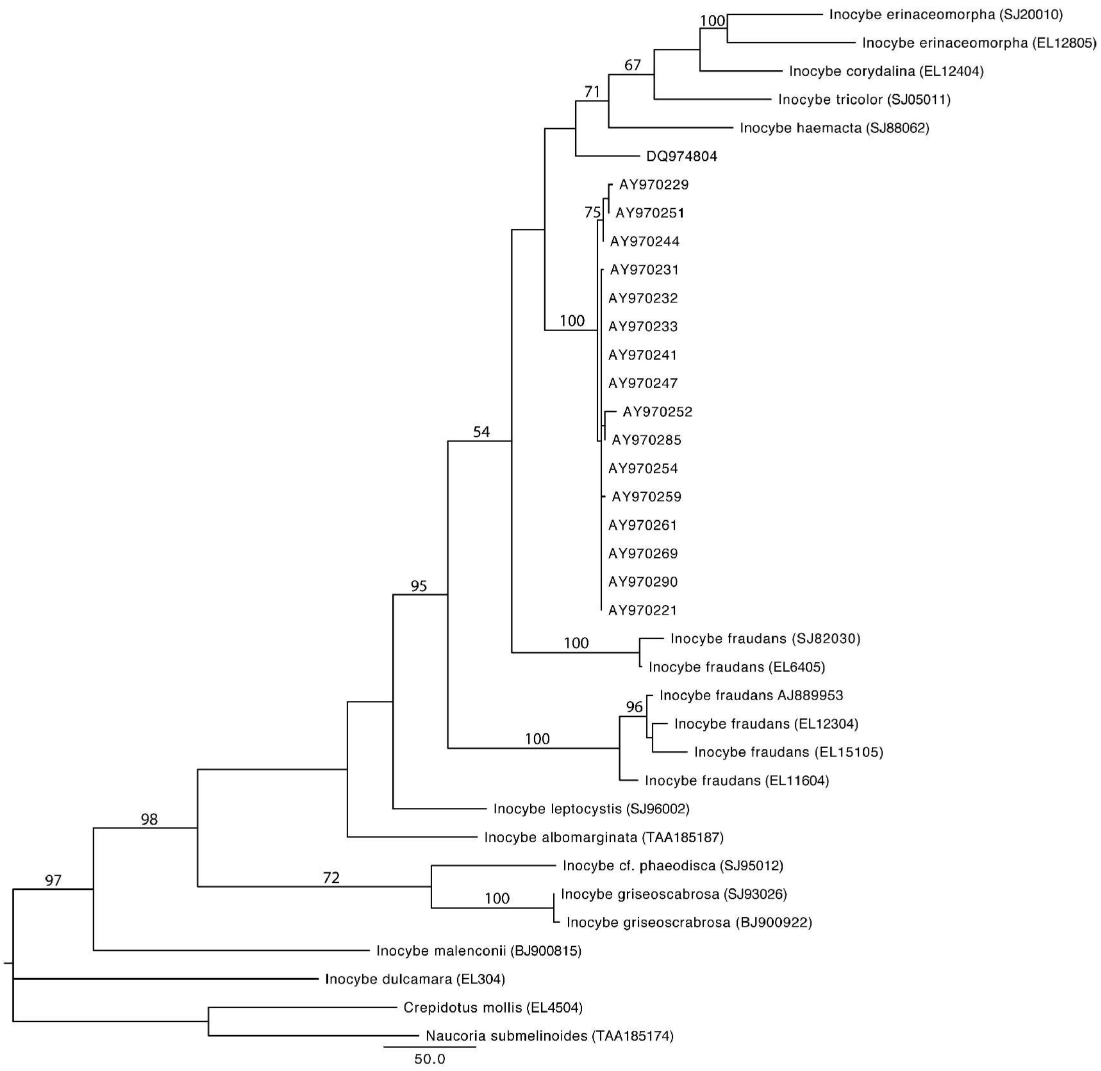

F. Alignment group 6. Sequences from Inocybe subgenus Inocybe. One of 2 most parsimonious trees. Alignment with 2232 characters of which 1507 were constant, 311 variable but parsimony-uninformative, and 414 were parsimony-informative. The tree is 1588 steps long. CI = 0.6581, RI = 0.6334.

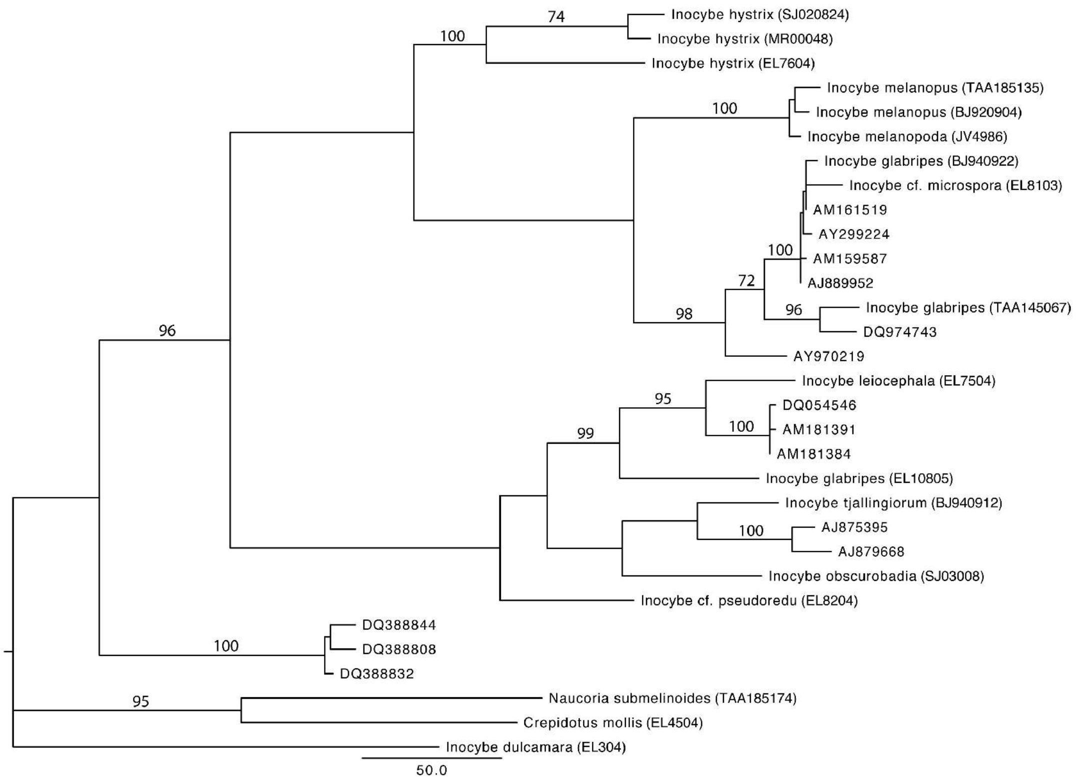

G. Alignment group 7. Sequences from Inocybe subgenus Inocybe. One of 562 most parsimonious trees. Alignment with 2263 characters of which 1579 were constant, 300 variable but parsimony-uninformative, and 384 were parsimony-informative. The tree is 1420 steps long. CI = 0.6859, RI = 0.7064.

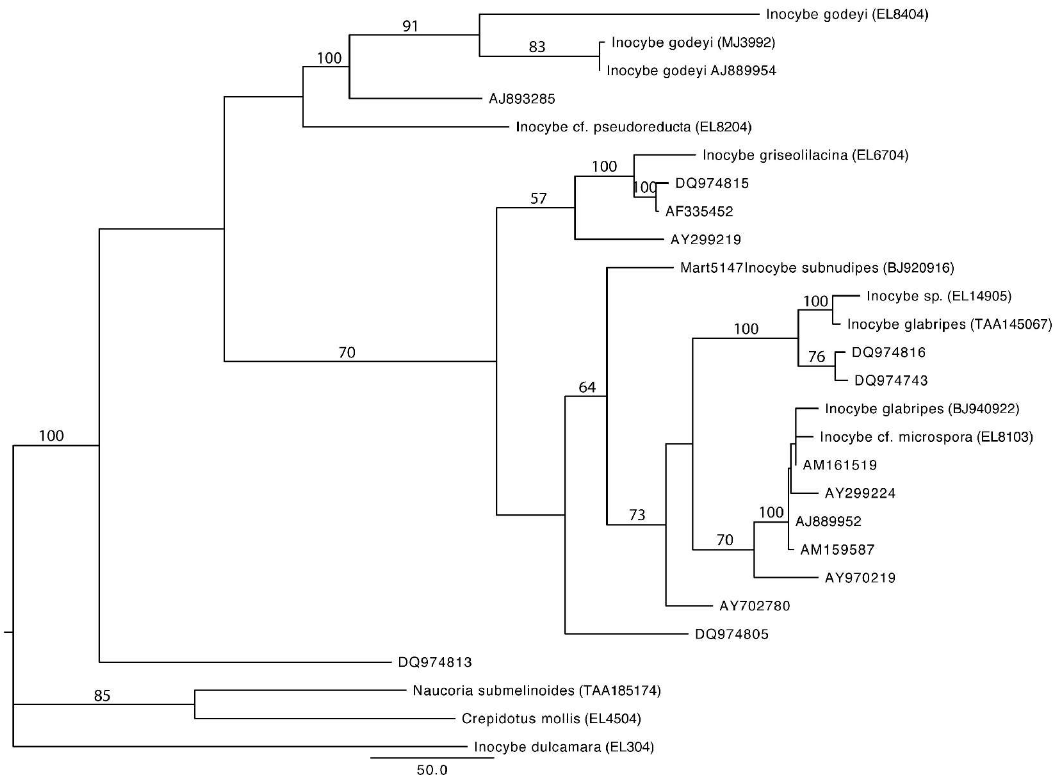

H. Alignment group 8. Sequences from Inocybe subgenus Inocybe. One of 45 most parsimonious trees. Alignment with 2256 characters of which 1571 were constant, 334 variable but parsimony-uninformative, and 351 were parsimony-informative. The tree is 1427 steps long. CI = 0.6804, RI = 0.6216.

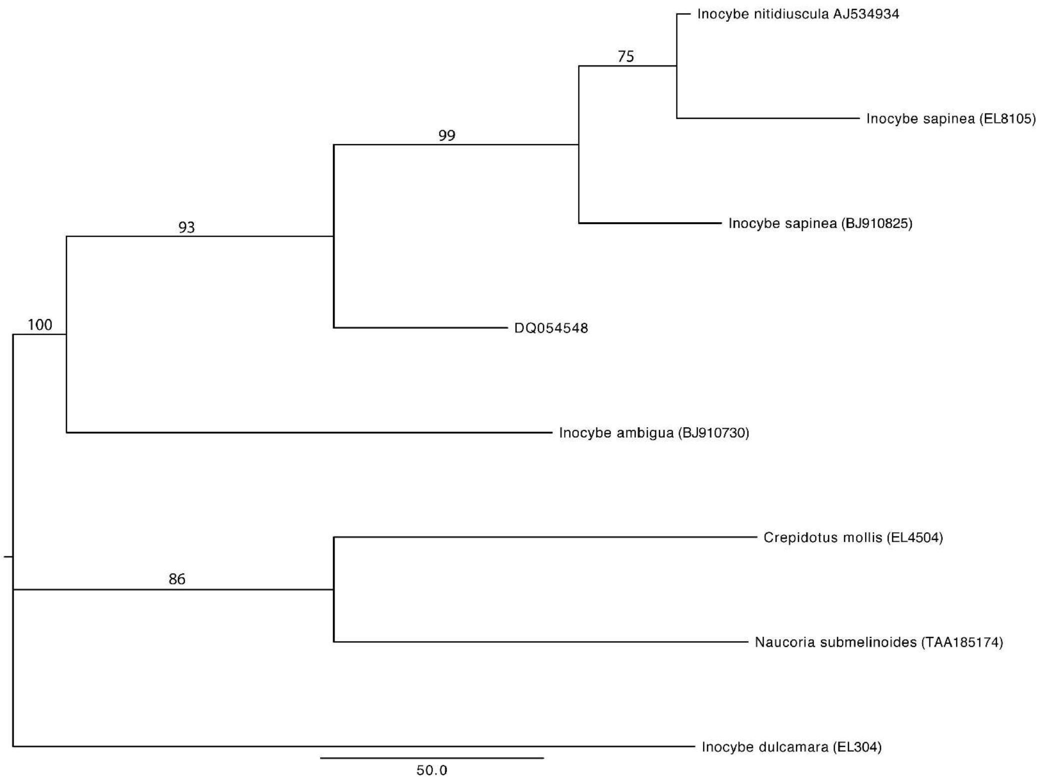

I. Alignment group 9. Sequences from Inocybe subgenus Inocybe. The most parsimonious tree. Alignment with 2163 characters of which 1691 were constant, 317 variable but parsimony-uninformative, and 155 were parsimony-informative. The tree is 627 steps long. CI = 0.9123, RI = 0.7381.

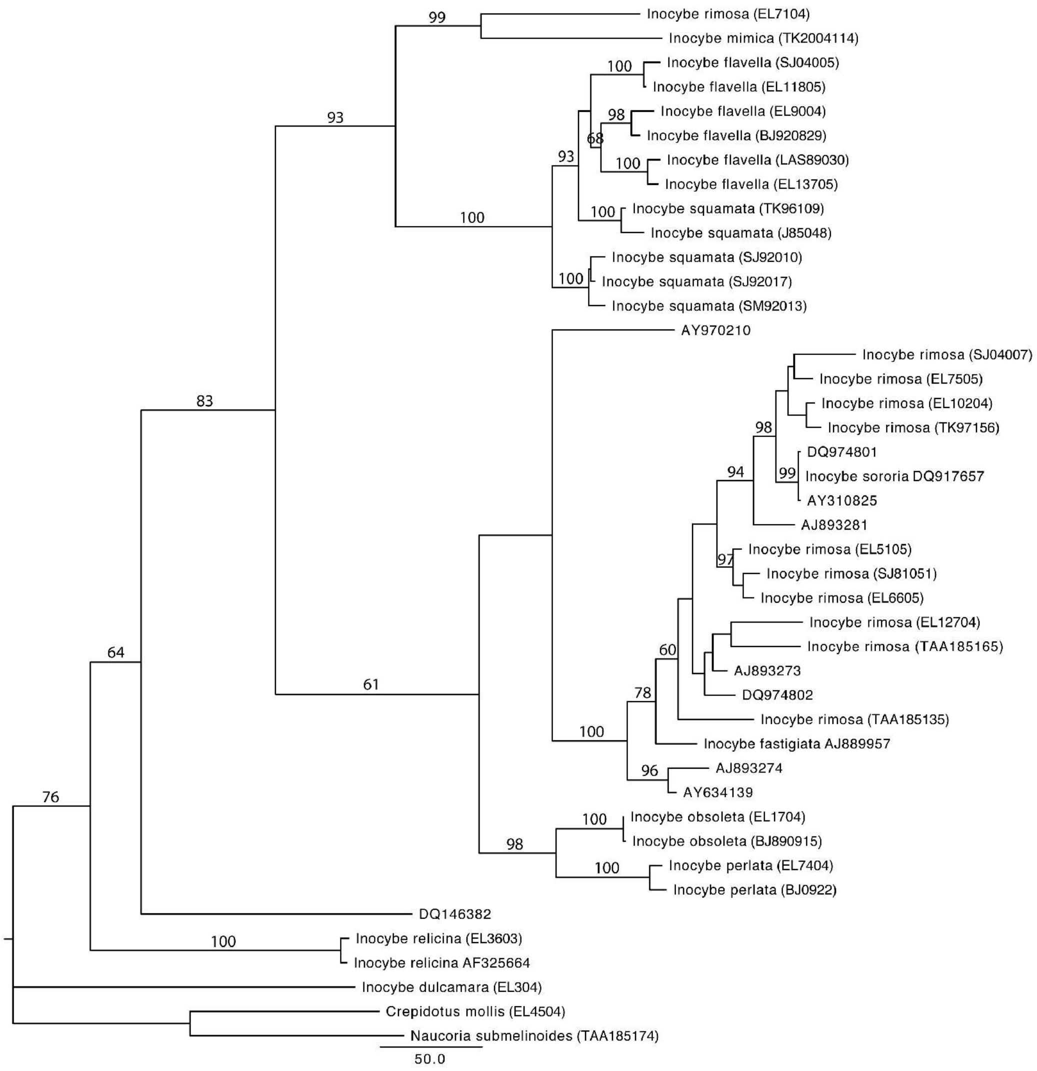

J. Alignment group 10. Sequences from Inocybe pseudosperma clade and I. relicina. One of 276 most parsimonious trees. Alignment with 2244 characters of which 1464 were constant, 318 variable but parsimony-uninformative, and 462 were parsimony-informative. The tree is 1872 steps long. CI = 0.6298, RI = 0.7275.

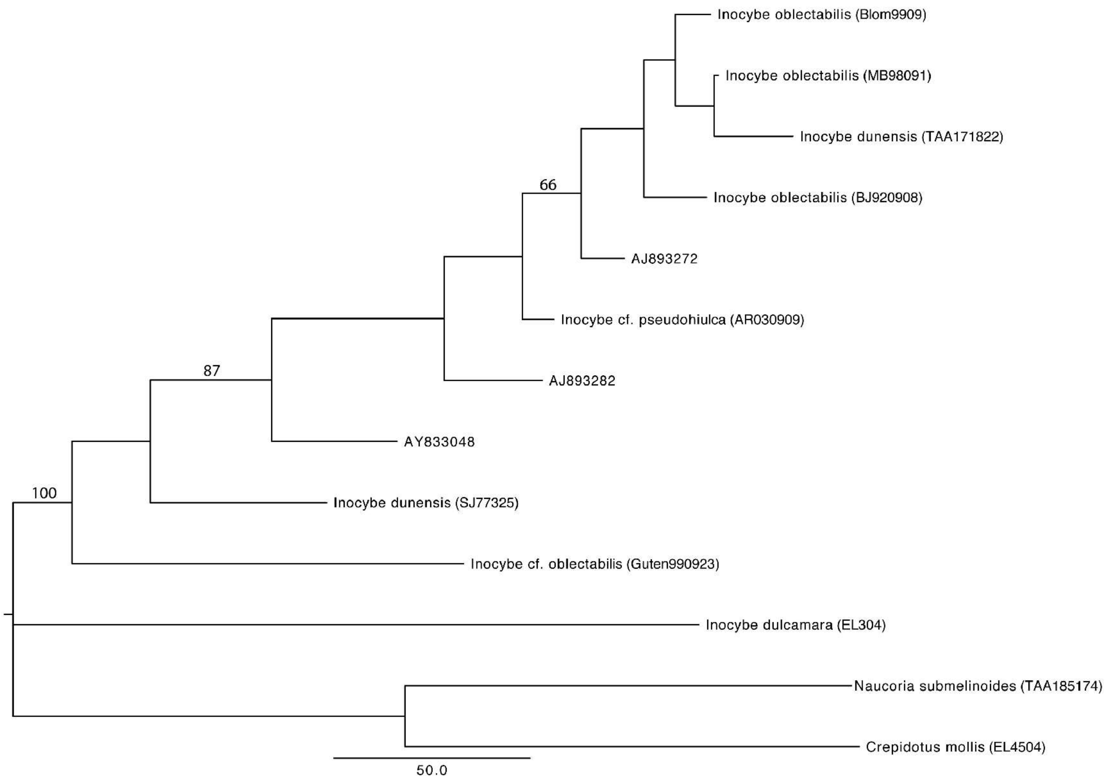

K. Alignment group 11. Sequences from Inocybe subgenus Inocybe. One of 10 most parsimonious trees. Alignment with 2166 characters of which 1620 were constant, 356 variable but parsimony-uninformative, and 190 were parsimony-informative. The tree is 763 steps long. CI = 0.9056, RI = 0.7608.

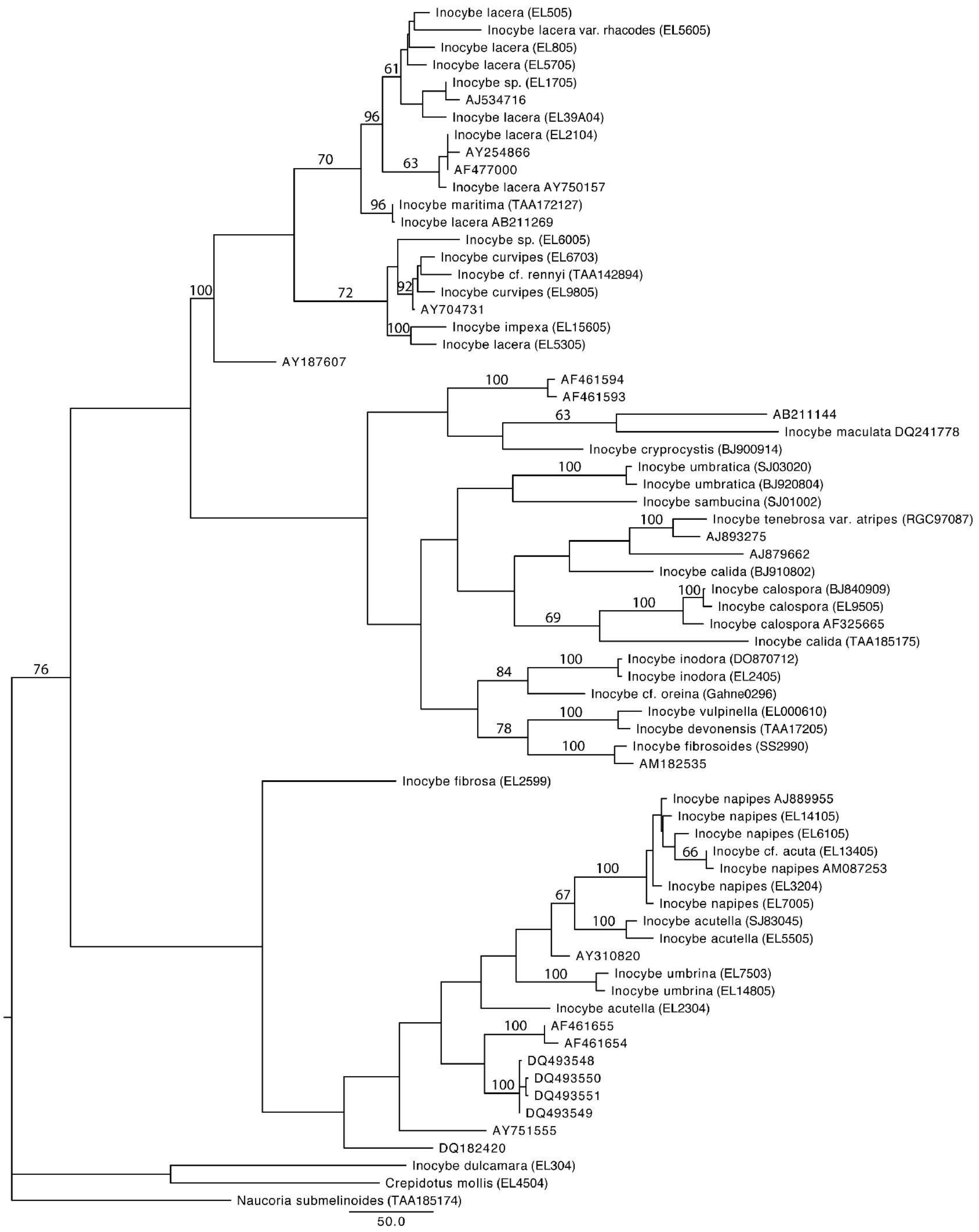

L. Alignment group 12. Sequences from Inocybe subgenus Inocybe. One of 420 most parsimonious trees. Alignment with 2272 characters of which 1301 were constant, 320 variable but parsimony-uninformative, and 651 were parsimony-informative. The tree is 3394 steps long. CI = 0.4782, RI = 0.6574.

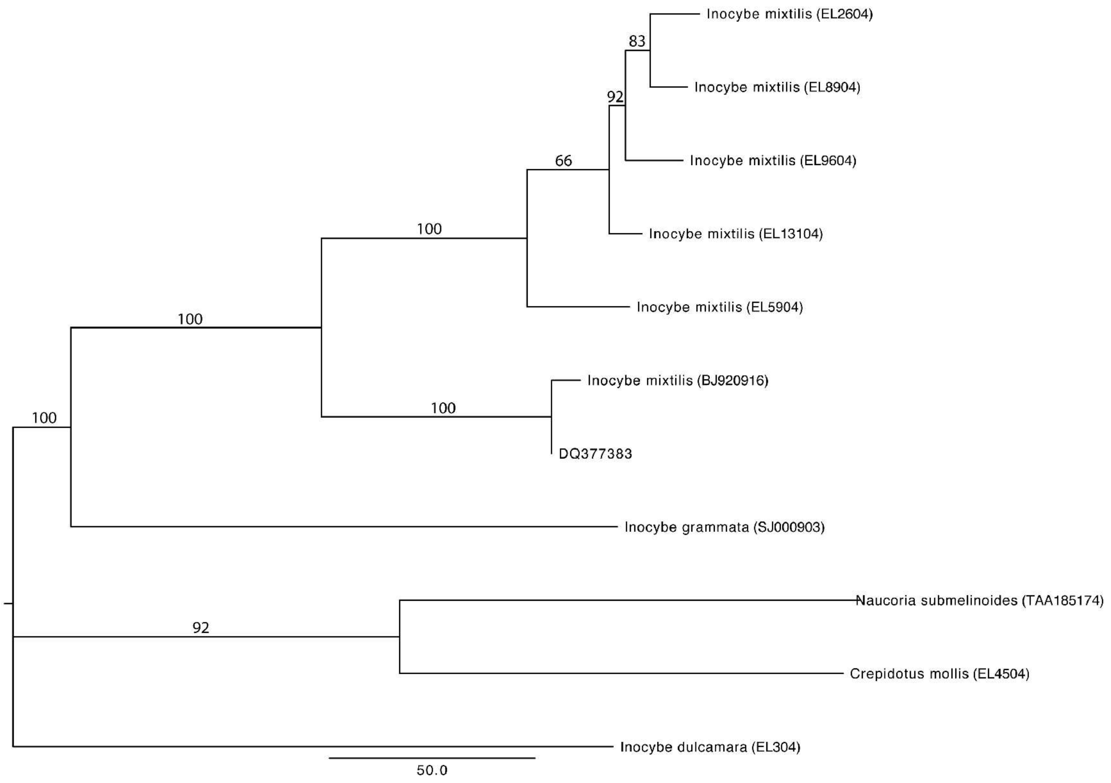

M. Alignment group 13. Sequences from Inocybe subgenus Inocybe. The most parsimonious tree. Alignment with 2170 characters of which 1655 were constant, 332 variable but parsimony-uninformative, and 183 were parsimony-informative. The tree is 715 steps long. CI = 0.8993, RI = 0.7517.

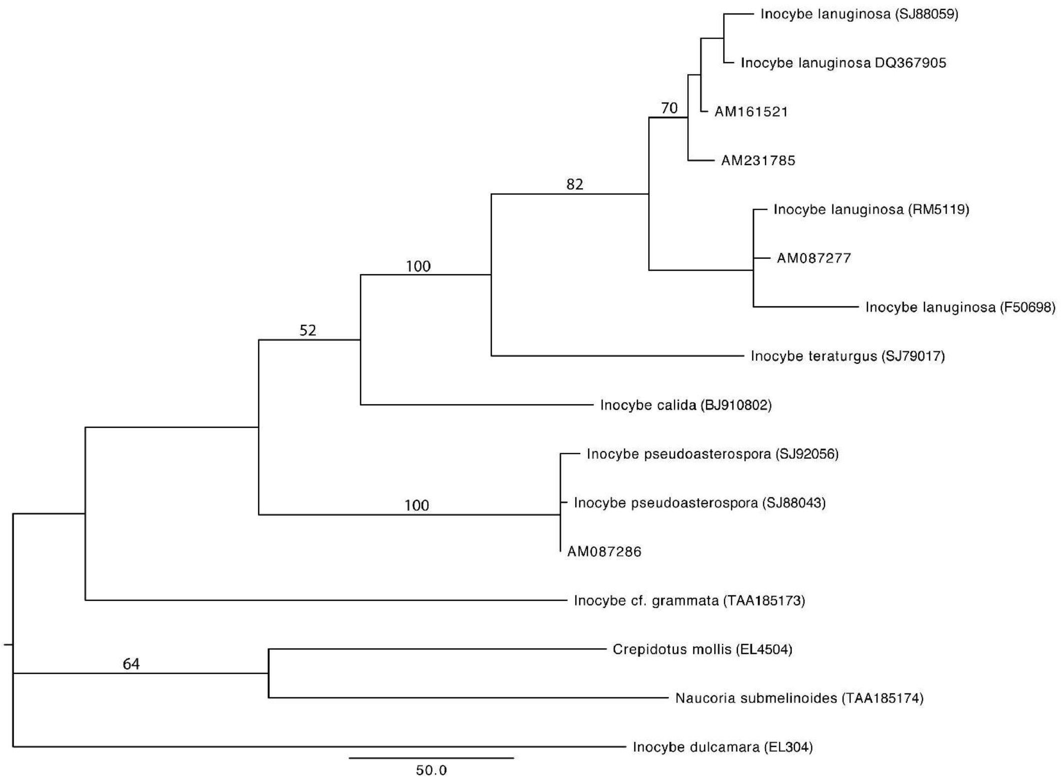

N. Alignment group 14. Sequences from Inocybe subgenus Inocybe. One of 60 most parsimonious trees. Alignment with 2197 characters of which 1601 were constant, 355 variable but parsimony-uninformative, and 241 were parsimony-informative. The tree is 960 steps long. CI = 0.6871, RI = 0.6700.

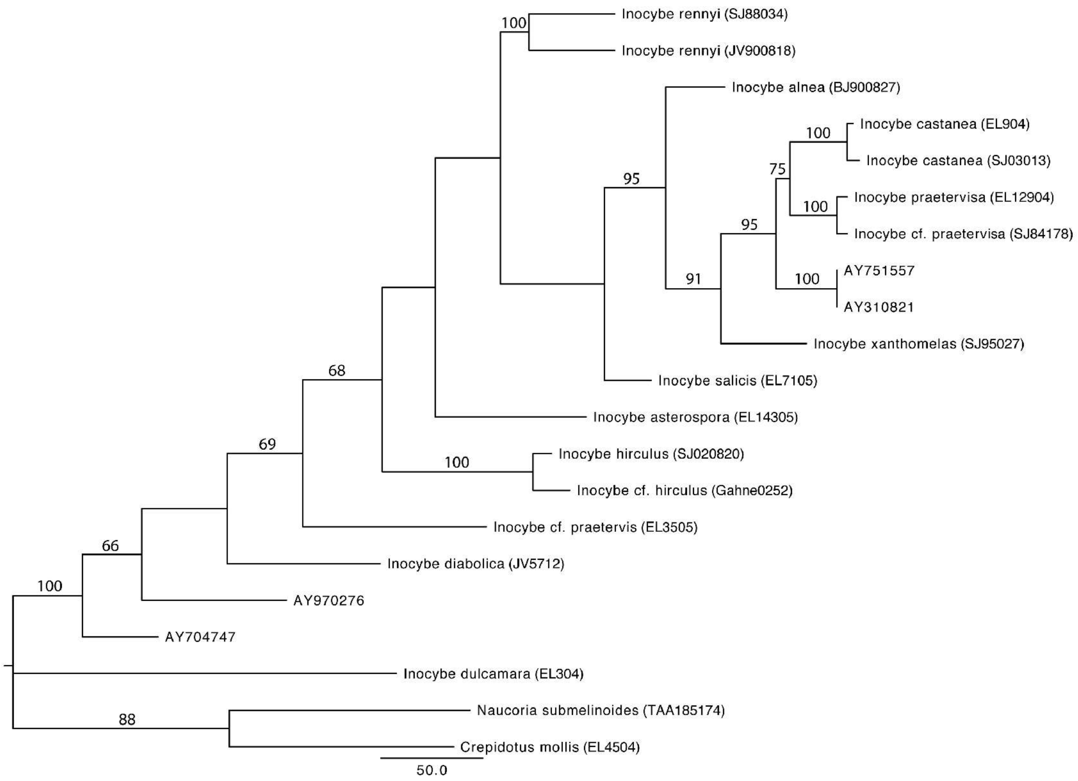

O. Alignment group 15. Sequences from Inocybe subgenus Inocybe. One of 2 most parsimonious trees. Alignment with 2220 characters of which 1557 were constant, 296 variable but parsimony-uninformative, and 367 were parsimony-informative. The tree is 1321 steps long. CI = 0.7010, RI = 0.5919.

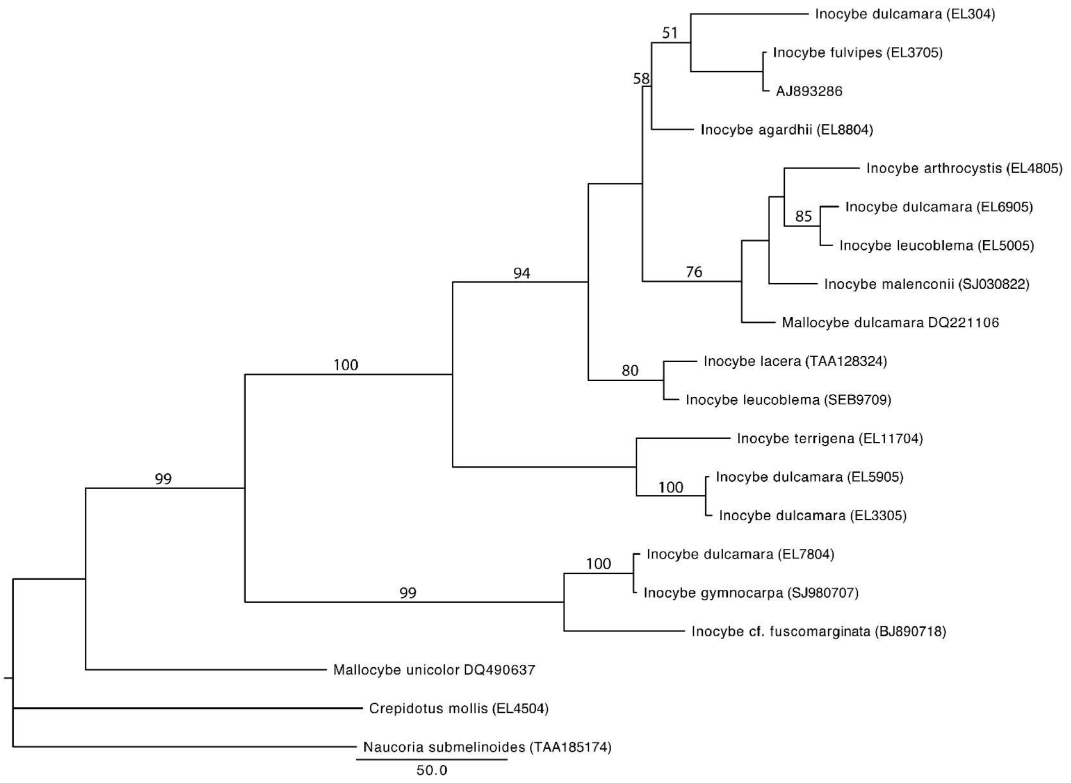

P. Alignment group 16. Sequences from Inocybe subgenus Mallocybe. One of 16 most parsimonious trees. Alignment with 2243 characters of which 1701 were constant, 307 variable but parsimony-uninformative, and 235 were parsimony-informative. The tree is 817 steps long. CI = 0.8201, RI = 0.7066.
