# Supplementary material for: Mining metadata from unidentified ITS sequences in GenBank: A case study in Inocybe (Basidiomycota)
Source: BMC Evol Biol. 2008 Feb 18;8:50. doi: 10.1186/1471-2148-8-50 (PMC2275786; doi:10.1186/1471-2148-8-50)
Supplement: Additional file 2 — Metadata associated with Inocybe sequences in GenBank. Unidentified (Table A) and fully identified (Table B) Inocybe sequences from GenBank included in this study; the studies they originate from (Table C); the ecology, as given in Kuyper [12], Stangl [13], and Hallingbäck and Aronsson [57], of the species they were determined to belong to (Table D); and sequences added in this study (Table E). [file 1471-2148-8-50-S2.pdf]

**Additional tables**

Unidentified (A) and identified (B) *Inocybe* sequences from GenBank included in this study; the studies they originate from (C); the ecology as given in Kuyper (1986), Stangl (1989), and Hallingbäck & Aronsson (2007) of the species they were determined to belong to (D); and sequences added in this study (E).

A)

| Accno | Species[1] | Taxa in study[2] | Host | Sub[3] | Litt[4] |
|---|---|---|---|---|---|
| AB096872 | *Inocybe aff. subporospora* | Unidentified | *Salix reinii* | root | 33 |
| AB211144 | *Inocybe sp. 51* | | | root | 53 |
| AB244041 | *Inocybe sp. 2* | *Inocybe sp.* | *Larix kaempferi* | root | 32 |
| AF335452 | *Inocybe aff. griseolilacina* | | | sporocarp | 3 |
| AF440670 | *Inocybe muricellata* | *Agaricales* | | root | 43 |
| AF440674 | *Inocybe hirtella* | *Bolbitiaceae* | | root | 43 |
| AF461593 | *Inocybe sp. 52* | Best BLAST *Inocybe cf. flocculosa* | | soil | 9 |
| AF461594 | *Inocybe sp. 52* | Best BLAST *Inocybe cf. flocculosa* | | soil | 9 |
| AF461654 | *Inocybe sp. 48* | Best BLAST *Omphalina grossula* | | soil | 9 |
| AF461655 | *Inocybe sp. 48* | Best BLAST *Omphalina grossula* | | soil | 9 |
| AF477000 | *Inocybe lacera* | Unidentified | | root | 40 |
| AF495458 | *Inocybe giacomi* | | *Kobresia mysoroides* | | 41 |
| AF495459 | *Inocybe giacomi* | | *Kobresia mysoroides* | | 41 |
| AJ534716 | *Inocybe lacera* | *Inocybe sp.* | *Betula pendula* | root | 47 |
| AJ534897 | *Inocybe cf. fuscidula* | *Inocybe sp.* | *Tilia cordata* | root | 47 |
| AJ534923 | *Inocybe cf. fuscidula* | *Inocybe sp.* | *Tilia cordata* | root | 47 |
| AJ633572 | *Inocybe aff. boltonii* | *Inocybe* | | root | 8 |

| Accno | Species[1] | Taxa in study[2] | Host | Sub[3] | Litt[4] |
|---|---|---|---|---|---|
| AJ633578 | *Inocybe aff. boltonii* | *Inocybe* | | root | 8 |
| AJ633585 | *Inocybe aff. boltonii* | *Inocybe* | | root | 8 |
| AJ633593 | *Inocybe aff. boltonii* | *Inocybe* | | root | 8 |
| AJ875395 | *Inocybe sp. 38* | *Homobasidio-mycetes* | *Phragmites australis* | Tisue sample | 34 |
| AJ879660 | *Inocybe aff. calida var. bruneorufa* | *Agaricales* | | root | 30 |
| AJ879662 | *Inocybe sp. 50* | *Agaricales* | | root | 30 |
| AJ879668 | *Inocybe sp. 37* | *Cortinariaceae* | | root | 30 |
| AJ879674 | *Inocybe sp. 50* | *Agaricales* | | root | 30 |
| AJ889952 | *Inocybe glabripes* | | | sporocarp | 21 |
| AJ893271 | *Inocybe quietiodor* | *Inocybe sp.* | | root | 49a,b |
| AJ893272 | *Inocybe oblectabilis* | *Inocybe sp.* | | root | 49a,b |
| AJ893273 | *Inocybe rimosa* | *Inocybe sp.* | | root | 49a |
| AJ893274 | *Inocybe aff. rimosa* | *Inocybe sp.* | | root | 49a,b |
| AJ893275 | *Inocybe aff. tenebrosa var. atripes* | *Inocybe sp.* | | root | 49a,b |
| AJ893276 | *Inocybe maculata* | *Inocybe sp.* | | root | 49a,b |
| AJ893277 | *Inocybe maculata* | *Inocybe maculata* | | root | 49a,b |
| AJ893278 | *Inocybe sp. 16* | *Inocybe sp.* | | root | 49a |
| AJ893279 | *Inocybe cf. boltonii* | *Inocybe sp.* | | root | 49a |
| AJ893280 | *Inocybe sp. 21* | *Inocybe sp.* | | root | 49b |
| AJ893281 | *Inocybe rimosa* | *Inocybe sp.* | | root | 49a,b |
| AJ893282 | *Inocybe oblectabilis* | *Inocybe sp.* | | root | 49b |
| AJ893283 | *Inocybe sp. 14* | *Inocybe sp.* | | root | 49a |
| AJ893284 | *Inocybe sp. 1* | *Inocybe sp.* | | root | 49a |
| AJ893285 | *Inocybe aff. godeyi* | *Inocybe sp.* | | root | 49a |
| AJ893286 | *Inocybe sp. 57* | *Inocybe sp.* | | root | 49a |
| AM087254 | *Inocybe aff. stellatospora* | | *Fagus sylvatica* | root | 13 |

| Accno | Species[1] | Taxa in study[2] | Host | Sub[3] | Litt[4] |
|---|---|---|---|---|---|
| AM087277 | *Inocybe lanuginosa* | | *Fagus sylvatica* | root | 13 |
| AM087286 | *Inocybe pseudoasterospora* | | *Fagus sylvatica* | root | 13 |
| AM159586 | *Inocybe petiginosa* | *Inocybe petiginosa* | | mesh bags | 20 |
| AM159587 | *Inocybe glabripes* | *Inocybe glabripes* | | mesh bags | 20 |
| AM161518 | *Inocybe asterospora* | *Inocybe asterospora* | *Fagus sylvatica* | root | 20 |
| AM161519 | *Inocybe glabripes* | *Inocybe glabripes* | *Fagus sylvatica* | root | 20 |
| AM161520 | *Inocybe petiginosa* | *Inocybe petiginosa* | *Fagus sylvatica* | root | 20 |
| AM161521 | *Inocybe lanuginosa* | *Inocybe sp.* | *Fagus sylvatica* | root | 20 |
| AM181384 | *Inocybe sp. 39* | *Inocybe sp.* | *Orthilia secunda* | root | 48 |
| AM181391 | *Inocybe sp. 39* | *Inocybe sp.* | *Orthilia secunda* | root | 48 |
| AM182533 | *Inocybe leptophylla* | *Inocybe sp.* | *Fagus sylvatica* | root | 20 |
| AM182535 | *Inocybe aff. fibrosoides* | *Inocybe sp.* | *Fagus sylvatica* | mesh bags | 20 |
| AM231783 | *Inocybe sp. 7* | | | root | 7 |
| AM231784 | *Inocybe sp. 7* | | | root | 7 |
| AM231785 | *Inocybe lanuginosa* | | | root | 7 |
| AY187607 | *Inocybe sp. 53* | *Cortinariaceae* | *Salix sp. (willow)* | root | 50 |
| AY254866 | *Inocybe lacera* | *Inocybe lacera* | | root | 45 |
| AY299219 | *Inocybe sp. 42* | *Agaricales* | | root | 6 |
| AY299224 | *Inocybe glabripes* | *Inocybe sp.* | | root | 6 |
| AY310819 | *Inocybe sp. 13* | *Cortinariaceae* | *Pseudotsuga* | root | 19 |
| AY310820 | *Inocybe sp. 49* | *Cortinariaceae* | *Pseudotsuga* and *Lithocarpus* | root | 19 |
| AY310821 | *Inocybe sp. 56* | *Cortinariaceae* | *Lithocarpus* | root | 19 |
| AY310822 | *Inocybe sp. 26* | *Cortinariaceae* | *Pseudotsuga* and *Lithocarpus* | root | 19 |
| AY310823 | *Inocybe maculata* | *Agaricales* | *Pseudotsuga* and *Lithocarpus* | root | 19 |
| AY310824 | *Inocybe sp. 23* | *Agaricales* | *Pseudotsuga* | root | 19 |

| Accno | Species[1] | Taxa in study[2] | Host | Sub[3] | Litt[4] |
|---|---|---|---|---|---|
| AY310825 | *Inocybe rimosa* | *Tricholomaceae* | *Pseudotsuga* and *Lithocarpus* | root | 19 |
| AY310826 | *Inocybe sp. 12* | *Agaricales* | *Pseudotsuga* and *Lithocarpus* | root | 19 |
| AY310827 | *Inocybe sp. 10* | *Agaricales* | *Pseudotsuga* | root | 19 |
| AY310828 | *Inocybe phaeocomis* | *Agaricales* | *Pseudotsuga* | root | 19 |
| AY310829 | *Inocybe sp. 8* | *Cortinariaceae* | *Pseudotsuga* and *Lithocarpus* | root | 19 |
| AY456377 | *Inocybe aff. boltonii* | Best BLAST *Inocybe nitidiuscula* | | soil | 12 |
| AY534209 | *Inocybe sp. 20* | *Agaricoid* | *Pseudotsuga menziesii* and *Tsuga heterophylla* | root | 14 |
| AY634114 | *Inocybe ochroalba* | *Inocybe* | *Epipactis dunensis* | root | 4c |
| AY634139 | *Inocybe aff. rimosa* | *Inocybe* | *Cephalanthera damasonium* | root | 4a |
| AY634140 | *Inocybe nitidiuscula* | *Inocybe* | *Epipactis atrorubens* | root | 4a |
| AY634142 | *Inocybe nitidiuscula* | *Inocybe* | *Pinus sylvestris* | root | 4b |
| AY702723 | *Inocybe sp. 32* | *Agaricoid* | | root | 17 |
| AY702724 | *Inocybe geophylla s.l.* | *Agaricoid* | | root | 17 |
| AY702725 | *Inocybe aff. splendens* | *Agaricoid* | | root | 17 |
| AY702727 | *Inocybe sp. 3* | *Agaricoid* | | root | 17 |
| AY702748 | *Inocybe sp. 29* | *Basidiomycota* | | root | 17 |
| AY702749 | *Inocybe geophylla s.l.* | *Basidiomycota* | | root | 17 |
| AY702780 | *Inocybe aff. glabirpes* | *Inocybe* | | root | 17 |
| AY704731 | *Inocybe curvipes* | *Basidiomycetes* | | soil | 55 |
| AY704747 | *Inocybe sp. 54* | *Hymenomycetes* | | soil | 55 |
| AY748859 | *Inocybe hirtella* | | *Salix caprea* | | 15 |
| AY750169 | *Inocybe sp. 29* | | *Pseudotsuga menziesii* | sporocarp | 10 |
| AY751555 | *Inocybe sp. 46* | | *Pseudotsuga menziesii* | sporocarp | 10 |
| AY751556 | *Inocybe sp. 8* | | *Pseudotsuga menziesii* | sporocarp | 10 |
| AY751557 | *Inocybe sp. 56* | | *Pseudotsuga menziesii* | sporocarp | 10 |

| Accno | Species[1] | Taxa in study[2] | Host | Sub[3] | Litt[4] |
|---|---|---|---|---|---|
| AY751558 | *Inocybe aff. alpigenes* | | *Pseudotsuga menziesii* | sporocarp | 10 |
| AY825511 | *Inocybe sp. 15* | *Cortinarius* | | root | 39 |
| AY825514 | *Inocybe sp. 18* | *Inocybe* | *Arbutus unedo (*maybe also *Quercus ilex)* | root | 39 |
| AY825515 | *Inocybe aff. flocculosa* | *Inocybe tigrina* | *Arbutus unedo (*maybe also *Quercus ilex)* | root | 39 |
| AY833048 | *Inocybe aff. oblectabilis* | *Cortinariaceae* | *Cephalanthera damasonium* | root | 18 |
| AY940653 | *Inocybe sp. 28* | Best BLAST match AF495459 | | root | 16 |
| AY969760 | *Inocybe sp. 31* | | | soil | 35a |
| AY969768 | *Inocybe sp. 31* | | | soil | 35a |
| AY969802 | *Inocybe sp. 24* | | | soil | 35a |
| AY969846 | *Inocybe sp. 31* | | | soil | 35a |
| AY969857 | *Inocybe sp. 11* | | | soil | 35a |
| AY969904 | *Inocybe sp. 31* | | | soil | 35a |
| AY970210 | *Inocybe sp. 44* | | | soil | 35b |
| AY970219 | *Inocybe glabripes* | | | soil | 35b |
| AY970221 | *Inocybe sp. 33* | | | soil | 35b |
| AY970229 | *Inocybe sp. 34* | | | soil | 35b |
| AY970231 | *Inocybe sp. 33* | | | soil | 35b |
| AY970232 | *Inocybe sp. 33* | | | soil | 35b |
| AY970233 | *Inocybe sp. 33* | | | soil | 35b |
| AY970241 | *Inocybe sp. 33* | | | soil | 35b |
| AY970244 | *Inocybe sp. 33 (or 34 based on ITS1)* | | | soil | 35b |
| AY970247 | *Inocybe sp. 33* | | | soil | 35b |
| AY970251 | *Inocybe sp. 34* | | | soil | 35b |
| AY970252 | *Inocybe sp. 33* | | | soil | 35b |
| AY970254 | *Inocybe sp. 33* | | | soil | 35b |
| AY970259 | *Inocybe sp. 33* | | | soil | 35b |
| AY970261 | *Inocybe sp. 33* | | | soil | 35b |
| AY970269 | *Inocybe sp. 33* | | | soil | 35b |
| AY970276 | *Inocybe sp. 55* | | | soil | 35b |
| AY970285 | *Inocybe sp. 33* | | | soil | 35b |

| Accno | Species[1] | Taxa in study[2] | Host | Sub[3] | Litt[4] |
|---|---|---|---|---|---|
| AY970290 | *Inocybe sp. 33* | | | soil | 35b |
| DQ054545 | *Inocybe sp. 2* | | | root | 52 |
| DQ054546 | *Inocybe sp. 39* | | | root | 52 |
| DQ054548 | *Inocybe aff. sapinea* | | | root | 52 |
| DQ054563 | *Inocybe sp. 31* | | | root | 52 |
| DQ054567 | *Inocybe sp. 17* | | | root | 52 |
| DQ054568 | *Inocybe sp. 19* | | | root | 52 |
| DQ054570 | *Inocybe cf. grammata* | | | root | 52 |
| DQ054573 | *Inocybe sp. 17* | | | root | 52 |
| DQ068957 | *Inocybe jacobi* | *Inocybe sp.* | *Picea abies* | root | 28 |
| DQ146372 | *Inocybe sp. 27* | | *Shorea obtusa* | root | 54 |
| DQ146375 | *Inocybe sp. 6* | | *Dipterocarpus turbinatus* | root | 54 |
| DQ146382 | *Inocybe sp. 43* | | *Hopea odorata* | root | 54 |
| DQ182420 | *Inocybe sp. 45* | | *Cephalanthera longifolia* | root | 42 |
| DQ182443 | *Inocybe cookei* | | *Cephalanthera longifolia* | root | 42 |
| DQ182447 | *Inocybe maculata* | | *Cephalanthera longifolia* | root | 42 |
| DQ182450 | *Inocybe sp. 22* | | *Cephalanthera longifolia* | root | 42 |
| DQ233747 | *Inocybe soluta* | | | root | 22 |
| DQ233757 | *Inocybe soluta* | | | root | 22 |
| DQ233761 | *Inocybe soluta* | | | root | 22 |
| DQ233793 | *Inocybe soluta* | | | root | 22 |
| DQ233794 | *Inocybe soluta* | | | root | 22 |
| DQ377383 | *Inocybe mixtilis* | | *Pinus taeda* | root | 37 |
| DQ377438 | *Inocybe phaeocomis* | | *Pinus taeda* | root | 37 |
| DQ388808 | *Inocybe sp. 36* | *Cortinariaceae* | | mesh bags | 1a,b |
| DQ388832 | *Inocybe sp. 36* | *Cortinariaceae* | | mesh bags | 1a |
| DQ388844 | *Inocybe sp. 36* | *Cortinariaceae* | | mesh bags | 1a |
| DQ388862 | *Inocybe sp. 25* | *Cortinariaceae* | | mesh bags | 1a |
| DQ493548 | *Inocybe sp. 47* | | | root | 51 |

| Accno | Species[1] | Taxa in study[2] | Host | Sub[3] | Litt[4] |
|---|---|---|---|---|---|
| DQ493549 | *Inocybe sp. 47* | | | root | 51 |
| DQ493550 | *Inocybe sp. 47* | | | root | 51 |
| DQ493551 | *Inocybe sp. 47* | | | root | 51 |
| DQ493569 | *Inocybe sp. 4* | | | root | 51 |
| DQ493570 | *Inocybe sp. 4* | | | root | 51 |
| DQ493587 | *Inocybe sp. 5* | | | root | 51 |
| DQ493588 | *Inocybe sp. 5* | | | root | 51 |
| DQ493589 | *Inocybe sp. 5* | | | root | 51 |
| DQ493590 | *Inocybe sp. 5* | | | root | 51 |
| DQ517413 | *Inocybe sp. 7* | | | | 29 |
| DQ517416 | *Inocybe jacobi* | | *Pinus albicaulis* | | 29 |
| DQ974741 | *Inocybe geophylla s.l. (whitei)* | *Inocybe cf. pudica* | *Quercus douglasii* | sporocarp | 46 |
| DQ974742 | *Inocybe sp. 9* | *Inocybe cf. pusio* | *Quercus douglasii* | sporocarp | 46 |
| DQ974743 | *Inocybe glabripes* | *Inocybe sp.* | *Quercus douglasii* | root | 46 |
| DQ974801 | *Inocybe rimosa* | *Inocybr cf. rimosa* | *Quercus douglasii* | sporocarp | 46 |
| DQ974802 | *Inocybe rimosa* | *Inocybe cf. sororia* | *Quercus douglasii* | sporocarp | 46 |
| DQ974804 | *Inocybe sp. 35* | Inocybe cf. fraudans | *Quercus douglasii* | sporocarp | 46 |
| DQ974805 | *Inocybe sp. 41* | *Inocybe cf. godeyi* | *Quercus douglasii* | sporocarp | 46 |
| DQ974806 | *Inocybe maculata* | *Inocybe cf. maculata* | *Quercus douglasii* | sporocarp | 46 |
| DQ974811 | *Inocybe maculata* | *Inocybe sp. (cf. lanatodisca)* | *Quercus douglasii* | root | 46 |
| DQ974812 | *Inocybe sp. 30* | *Inocybe sp. (cf. sindonia)* | *Quercus douglasii* | root | 46 |
| DQ974813 | *Inocybe sp. 40* | *Inocybe sp.* | *Quercus douglasii* | root | 46 |
| DQ974814 | *Inocybe maculata* | | *Quercus douglasii* | root | 46 |
| DQ974815 | *Inocybe aff. griseolilacina* | *Inocybe sp. (cf. flocculosa)* | *Quercus douglasii* | root | 46 |
| DQ974816 | *Inocybe glabripes* | *Inocybe sp.* | *Quercus douglasii* | root | 46 |

[1]*aff.* refers to species that are neighboring fully identified sequences while *cf.* refers to sequences assigned to the same species as an unidentified sequence added in this work. [2]The taxonomic annotation given to the sequence in the paper it originated from. [3]Sample substrate, [4]Corresponding to nr. in Table C.

B.

| Accno | Voucher (as given in GenBank) | Species | Litt[1] |
|---|---|---|---|
| DQ974803 | SRC225 | *Inocybe armeniaca* | 46 |
| DQ917657 | SRC514 | *Inocybe sororia* | 27 |
| AM087253 | | *Inocybe napipes* | 13 |
| AY750157 | C25 EC175 | *Inocybe lacera* | 10 |
| AB244791 | FORBs04003 | *Inocybe nitidiuscula* | 36 |
| AJ889957 | TF01-031 | *Inocybe fastigiata* | 21 |
| AJ889956 | MC01-508 | *Inocybe petiginosa* | 21 |
| AJ889955 | MC01-516 | *Inocybe napipes* | 21 |
| AJ889954 | TF01-025 | *Inocybe godeyi* | 21 |
| AJ889953 | TF01-037 | *Inocybe fraudans* | 21 |
| AJ889951 | TF01-038 | *Inocybe asterospora* | 21 |
| AJ889950 | MC01-530 | *Inocybe asterospora* | 21 |
| DQ490637 | RV7/4 | *Mallocybe unicolor* | 24 |
| AM113952 | JV99-370 | *Inocybe petiginosa* | 20 |
| DQ404391 | | *Inocybe cookei* | 25 |
| DQ404390 | | *Inocybe asterospora* | 25 |
| DQ367905 | | *Inocybe lanuginosa var. lanuginosa* | 11 |
| DQ093854 | | *Inocybe geophylla* | 11 |
| AB211269 | | *Inocybe lacera* | 31 |
| DQ241778 | | *Inocybe maculata* | 25 |
| DQ221106 | JV19652F | *Mallocybe dulcamara* | 26 |
| DQ067580 | CMI-UNIBO 5033 | *Inocybe splendens* | 5 |
| DQ067579 | CMI-UNIBO 5037 | *Inocybe rufuloides* | 5 |
| AJ966749 | | *Inocybe maculata* | 49 |
| AF347101 | EL 27-99 | *Inocybe cervicolor* | 23 |
| AJ534933 | TAA-185038 | *Inocybe maculata* | 47 |
| AJ534934 | TAA-185039 | *Inocybe nitidiuscula* | 47 |
| AY228354 | UBC F14289 | *Inocybe flocculosa* | 2 |
| AY228341 | UBC F14307 | *Inocybe pudica* | 44 |
| AF325665 | IB 19951008 | *Inocybe calospora* | 38 |
| AF325664 | IB 19920112 | *Inocybe relicina* | 38 |

[1] Corresponding to nr. in Table C.

C.

| Nr. | Comment | References |
| --- | --- | --- |
| 1a | *Eucalyptus pilularis* dominated wet sclerophyll forest burned every 4th-year. | Bastias BA, Xu Z, Cairney JWG. 2006. Influence of long-term repeated prescribed burning on mycelial communities of ectomycorrhizal fungi. New Phytologist 172: 149-158. |
| 1b | *Eucalyptus pilularis* dominated wet sclerophyll forest, control plot not burned. | Bastias,BA, Xu Z, Cairney JWG. 2006. Influence of long-term repeated prescribed burning on mycelial communities of ectomycorrhizal fungi. New Phytologist 172: 149-157. |
| 2 | | Beach A, Berbee ML, Inderbitzin P, Fischer AL. DirectSubmission Submitted (03-FEB-2003), class project. |
| 3 | | Berbee ML, Inderbitzin P, Zhang G. Class project, Structure and Reproduction of Fungi. Unpublished. |
| 4a | Open *Pinus sylvestris* forest with some *Picea abies* and *Fagus sylvatica*. On soil originating from dolomite. | Bidartondo MI, Burghardt B, Gebauer G, Bruns TD, Read DJ. 2004. Changing partners in the dark: isotopic and molecular evidence of ectomycorrhizal liaisons between forest orchids and trees. Proceedings of the Royal Society of London B 271: 1799-1806. |
| 4b | Open mixed stand with *Pinus sylvestris* and *Quercus robur*. On soil originating from dolomite. | Bidartondo MI, Burghardt B, Gebauer, G, Bruns TD, Read DJ. 2004. Changing partners in the dark: isotopic and molecular evidence of ectomycorrhizal liaisons between forest orchids and trees. Proceedings of the Royal Society of London B 271: 1799-1805. |
| 4c | *Pinus*-forested dunes. | Bidartondo MI, Burghardt B, Gebauer G, Bruns TD, Read DJ. 2004. Changing partners in the dark: isotopic and molecular evidence of ectomycorrhizal liaisons between forest orchids and trees. Proceedings of the Royal Society of London B 271: 1799-1804. |
| 5 | | Bonuso E, Iotti M, Zambonelli A. Inocybe spp. collected in cultivated truffieres. Unpublished. |
| 6 | Pure, 80-year-old *Fagus sylvatica* forest, thinned and unthinned, on clay from limestone. | Buee M, Vairelles D, Garbaye J. 2005. Year-round monitoring of diversity and potential metabolic activity of the ectomycorrhizal community in a beech (*Fagus silvatica*) forest subjected to two thinning regimes. Mycorrhiza 15: 235-245. |
| 7 | | Burke DJ, Kretzer AM. Molecular analysis of bacterial communities associated with the roots of Douglas fir (*Pseudotsuga menziesii*) colonized by different ectomycorrhizal fungi. Unpublished. |

| Nr. | Comment | References |
| --- | --- | --- |
| 8 | 8-year-old *Pinus taeda* plantation. | Burke DJ, Martin KJ, Rygiewicz PT, Topa MA. 2005. Ectomycorrhizal fungi identification in single and pooled root samples: terminal restriction fragment length polymorphism (TRFLP) and morphotyping compared. Soil Biology & Biochemistry 37: 1683-1694. |
| 9 | Open sclerophyll forest, *Eucalyptus ssp.* among others. | Chen DM, Cairney JWG. 2002. Investigation of the influence of prescribed burning on ITS profiles of ectomycorrhizal and other soil fungi at three Australian sclerophyll forest sites. Mycolgical Research 106: 532-540. |
| 10 | | Cline E. Mycorrhizal fungi communities of Douglas-fir (*Pseudotsuga menziesii*) seedlings and trees: effects of proximity to residual trees. Thesis (2004). |
| 11 | | Durall DM, Gamiet S, Simard SW, Kudrna L, Sakakibara SM. 2006. Effects of clearcut logging and tree species composition on the diversity and community composition of epigeous fruitbodies formed by ectomycorrhizal fungi. Canadian Journal of Botany 84: 966-980. |
| 12 | *Pinus taeda* plantation on welldrained sandy soil, fertilized and non fertilized. | Edwards IP, Cripliver JL, Gillespie AR, Johnsen KH, Scholler M, Turco RF. 2004. Nitrogen availability alters macrofungal basidiomycete community structure in optimally fertilized loblolly pine forests. New Phytologist 162: 755-770. |
| 13 | | Fons K, Kjoller R. The influence of forest continuity and geographic distance on the ectomycorrhizal fungal community associated with beech. Unpublished. |
| 14 | 40-50-year-old *Pseudotsuga menziesii* forest on soil from sandstones and mudstones. | Horton TR, Molina R, Hood K. 2005. Douglas-fir ectomycorrhizae in 40- and 400-year-old stands: mycobiont availability to late successional western hemlock. Mycorrhiza 15: 393-403. |
| 15 | | Hrynkiewicz K, Baum C, Haug I. Morphological and molecular characterization of ectomycorrhizas of *Salix caprea* at heavy metal polluted and control sites. Unpublished. |
| 16 | Broad leaved forest. | Iotti M, Zambonelli A. 2006. A quick and precise technique for identifying ectomycorrhizas by PCR. Mycolgical Research 110: 60-65. |

| Nr. | Comment | References |
|---|---|---|
| 17 | Old-growth mixed-conifer forest with *Abies concolor* and *A. magnifica* as dominants but also *Pinus jeffreyi, P. lambertiana,* and *Calocedrus decurrens*. Coarse and loamy soil. | Izzo A, Agbowo J, Bruns TD. 2005. Detection of plot-level changes in ectomycorrhizal communities across years in an old-growth mixed-conifer forest. New Phytologist 166: 619-629. |
| 18 | *Quercus robur* and *Corylus avellana* forest on calcareous soil. | Julou T, Burghardt B, Gebauer G, Berveiller D, Damesin C, Selosse MA. 2005. Mixotrophy in orchids: insights from a comparative study of green individuals and nonphotosynthetic individuals of *Cephalanthera damasonium*. New Phytologist 166: 639-653. |
| 19 | Mixed evergreen forest dominated by *Pseudotsuga menziesii* but also with *Lithocarpus densiflora, Quercus agrifolia, Q. chrysolepis, Arbutus menziesii, Umbelluaria californica* and *Sequoia sempervirens*. | Kennedy PG, Izzo AD, Bruns TD. 2003. There is high potential for the formation of common mycorrhizal networks between understory and canopy trees in a mixed evergreen forest. Journal of Ecology 91: 1071-1080. |
| 20 | 80-year-old *Fagus sylvatica* forest. | Kjoller R. 2006. Disproportionate abundance between ectomycorrhizal root tips and their associated mycelia. FEMS Microbiology Ecology 58: 214-224. |
| 21 | | Kjoller R. ITS sequences of ectomycorrhizal fungi from beech forests. Unpublished. |
| 22 | *Picea abies* field trial plantation on podzolized, fine sand moraine soil. | Korkama T, Pakkanen A, Pennanen T. 2006. Ectomycorrhizal community structure varies among Norway spruce (*Picea abies*) clones. New Phytologist 171, 815-824. |
| 23 | | Larsson K-H, Larsson E, Koljalg U. 2004. High phylogenetic diversity among corticioid homobasidiomycetes. Mycological Research 108: 983-1002. |
| 24 | | Matheny PB, Curtis JM, Hibbett DS. Six major clades of *Agaricales*. Unpublished. |
| 25 | | Matheny PB, Hibbett DS. Assembling the Fungal Tree of Life. Unpublished. |

| Nr. | Comment | References |
| --- | --- | --- |
| 26 | | Matheny PB, Wang Z, Binder M, Curtis JM, Lim YW, Nilsson RH, Hughes KW, Hofstetter V, Ammirati JF, Schoch CL, Langer E, Langer G, McLaughlin DJ, Wilsona AW, Frøslevj T, Ge Z-W, Kerrigan RW, Slot JC, Yang Z-L, Baroni TJ, Fischer M, Hosaka K, Matsuura K, Seidl MT, Vauras J, Hibbett DS. In press. Contributions of rpb2 and tef1 to the phylogeny of mushrooms and allies (*Basidiomycota*, *Fungi*). Molecular Phylogenetics and Evolution. |
| 27 | | Matheny PB. Direct Submission Submitted (24-AUG-2006). |
| 28 | From plant nursery. | Menkis A, Vasiliauskas R, Taylor AFS, Stenlid J, Finlay R. 2005. Fungal communities in mycorrhizal roots of conifer seedlings in forest nurseries under different cultivation systems, assessed by morphotyping, direct sequencing and mycelial isolation. Mycorrhiza 16: 33-41. |
| 29 | | Mohatt KR, Cripps CL. Ectomycorrhizal fungi of whitebark pine in the northern Greater Yellowstone Ecosystem. Unpublished. |
| 30 | Truffle ground with up to 50 year old trees, e.g. *Quercus ssp, Populus spp., Salix alba,* and *Tilia cordata*. | Murat C, Vizzini A, Bonfante P, Mello A. 2005. Morphological and molecular typing of the below-ground fungal community in a natural *Tuber magnatum* truffle-ground. FEMS Microbiology Letters 245: 307-313. |
| 31 | | Nara K. 2006. Ectomycorrhizal networks and seedling establishment during early primary succession. New Phytologist 169: 169-178. |
| 32 | Volcanic desert. | Nara K. 2006. Pioneer dwarf willow may facilitate tree succession by providing late colonizers with compatible ectomycorrhizal fungi in a primary successional volcanic desert. New Phytologist 171: 187-197. |
| 33 | | Nara K. Ectomycorrhizal fungal communities during early primary succession on Mt. Fuji. Published Only in Database (2002). |
| 34 | Lake shore. | Neubert K, Mendgen K, Brinkmann H, Wirsel SGR. 2006. Only a few fungal species dominate highly diverse mycofloras associated with the common reed. Applied Environmental Microbiology 72: 1118-1128. |

| Nr. | Comment | References |
|---|---|---|
| 35a | Early successional *Pinus taeda* stand on well drained land with a few hardwoods in the understory. | O'brien HE, Parrent JL, Jackson JA, Moncalvo J-M, Vilgalys R. 2005. Fungal community analysis by large-scale sequencing of environmental samples. Applied Environmental Microbiology 71: 5544-5550. |
| 35b | Late successional mixed hardwood stand on alluvial soil. | O'brien HE, Parrent JL, Jackson JA, Moncalvo J-M, Vilgalys R. 2005. Fungal community analysis by large-scale sequencing of environmental samples. Applied Environmental Microbiology 71: 5544-5550. |
| 36 | | Obase K, Kobayashi T, Miyamoto T, Tamai Y, Yajima T. 2006. *Inocybe nitidiuscula*, new to Japan. Mycoscience 47: 293-297. |
| 37 | 17-year-old *Pinus taeda* stand on somewhat acidic clay rich soil. | Parrent JL, Morris WF, Vilgalys R. 2006. CO2-enrichment and nutrient availability alter ectomycorrhizal fungal communities. Ecology 87: 2278-2287. |
| 38 | | Peintner U, Bougher NL, Castellano MA, Moncalvo J-M, Moser MM, Trappe JM, Vilgalys R. 2001. Multiple origins of sequestrate fungi related to *Cortinarius* (*Cortinariaceae*). American Journal of Botany 88: 2168-2179. |
| 39 | *Quercus ilex* forest with understory (chaparral) with *Phillyrea latifolia*, *Erica arborea* and *Arbutus unedo*. On slightly acid soil. | Richard F, Millot S, Gardes M, Selosse M-A. 2005. Diversity and specificity of ectomycorrhizal fungi retrieved from an old-growth Mediterranean forest dominated by *Quercus ilex*. New Phytologist 166: 1011-1023. |
| 40 | Coniferous forest on podzol soil profile, *Picea abies* and *Pinus sylvestris*. | Rosling A, Landeweert R, Lindahl B, Larsson K-H, Kuyper TW, Taylor AFS, Finlay RD. 2003. Vertical distribution of ectomycorrhizal fungal taxa in a podzol soil profile. New Phytologist 159: 775-783. |
| 41 | | Schadt CW, Schmidt SK. Isolation, characterization and phylogenetic identification of the dominant ectomycorrhizal fungi associated with the alpine sedge *Kobresia myosuroides*. Unpublished. |
| 42 | | Selosse M-A. *Cephalanthera longifolia* mycorrhizal fungi. Unpublished. |
| 43 | Ectomycorrhiza intermingled with roots of *Neottia nidus-avis*. | Selosse M-A, Weiss M, Jany J-L, Tillier A. 2002. Communities and populations of sebacinoid basidiomycetes associated with the achlorophyllous orchid *Neottia nidus-avis* (L.) L.C.M. Rich. and neighbouring tree ectomycorrhizae Molecular Ecology 11: 1831-1844. |

| Nr. | Comment | References |
|---|---|---|
| 44 | | Skogstad D, Berbee ML, Inderbitzin P, Fischer AL. Direct Submission Submitted (03-FEB-2003) class project. |
| 45 | 75-year-old *Pinus sylvestris* forest with litter removed. | Smit E, Veenman C, Baar J. 2003. Molecular analysis of ectomycorrhizal basidiomycete communities in a *Pinus sylvestris* L. stand reveals long-term increased diversity after removal of litter and humus layers. FEMS Microbiology Ecology 45: 49-57. |
| 46 | Site dominated by *Quercus douglasii* but *Q. wislizeni* and *Pinus sabiniana* also present. | Smith ME, Douhan GW, Rizzo DM. Ectomycorrhizal community structure in a xeric *Quercus* woodland based on rDNA sequence analysis of sporocarps  and pooled roots. New Phytologist 174: 847-863. |
| 47 | Mixed forest dominated by *Picea abies* and *Tilia cordata* but also a few *Betula pendula, Populus tremula*  and *Sorbus aucuparia*. On podzol soil. | Tedersoo L, Koljalg U, Hallenberg N, Larsson K-H. 2003. Fine scale distribution of ectomycorrhizal fungi and roots across substrate layers including coarse woody debris in a mixed forest. New Phytologist 159: 153-165. |
| 48 | 100-120 year-old *Pinus sylvestris* forest with sparse *Picea abies* undergrowth, podzol profile on sand. | Tedersoo L, Pellet P, Koljalg U, Selosse M-A. 2006. Parallel evolutionary paths to mycoheterotrophy in understorey *Ericaceae* and *Orchidaceae*: ecological evidence for mixotrophy in *Pyroleae*. Oecologia 151: 206-217. |
| 49a | Forested former meadow, several broad leafed trees. | Tedersoo L, Suvi T, Larsson E, Koljalg U. 2006. Diversity and community structure of ectomycorrhizal fungi in a wooded meadow. Mycological Research 110:  734-748. |
| 49b | Wooded meadow, several broad leafed trees. | Tedersoo L, Suvi T, Larsson E, Koljalg U. 2006. Diversity and community structure of ectomycorrhizal fungi in a wooded meadow. Mycological Research 110:  734-748. |
| 50 | Forefront of receding glacier. | Trowbridge J, Jumpponen A. 2004. Fungal colonization of shrub willow roots at the forefront of a receding glacier. Mycorrhiza 14: 283-293. |
| 51 | | Wilson AW, Hobbie EA. Hibbett DS. The ectomycorrhizal status of *Calostoma cinnabarinum* using isotopicand molecular methods. Unpublished. |
| 52 | | Wilson MJ, Certini G, Campbell CD, Anderson IC, Hillier S, Fraser AR, Delbos E, McMurray E. Mineral weathering and ectomycorrhizal fungi: are they speleologists or miners? Unpublished. |

| Nr. | Comment | References |
|---|---|---|
| 53 | | Wu B, Zhou Z and Hogetsu T. Ectomycorrhizal fungi colonizing *Pinus densiflora*. Published Only in Database (2005). |
| 54 | *Dipterocarpaceae* forest. | Yuwa-amornpitak T, Vichitsoonthonkul T, Tanticharoen M, Cheevadhanarak S, Ratchadawong S. 2006. Diversity of Ectomycorrhizal Fungi on *Dipterocarpaceae* in Thailand. Journal of Biological Sciences 6: 1059-1064. |
| 55 | Silty-loam soil treated with fertilizer and incubated. | Zhao Y, Wu L, Zhou Z, Wang L, Pan Y, Zhao L. 2005. Dynamics of microbial community structure and cellulolytic activity in agricultural soil amended with two biofertilizers. European Journal of Soil Biology 41: 21-29. |

D.

| Species | Ecology |
|---|---|
| *Inocybe asterospora* | Broadleaved forest and parks on nutrient rich, calciferous soil. |
| *Inocybe cookei* | Broadleaved and mixed forest. |
| *Inocybe curvipes* | With broadleaved trees at forest edges, parks and gardens. |
| *Inocybe geophylla s.l.* | On nutrient rich soil, mostly with broadleaved trees, rarely with coniferous trees. |
| *Inocybe glabripes* | With broadleaved trees preferably in parks but also in natural vegetation. |
| *Inocybe hirtella* | With broadleaved trees on calciferous soil. |
| *Inocybe jacobi* | Somewhat contradictory accounts. With *Picea* or with *Pinus* on sandy soil. |
| *Inocybe lacera* | With broadleaved and/or coniferous trees on nutrient poor soils. |
| *Inocybe lanuginosa* | In coniferous forest on somewhat acidious soil, often with *Sphagnum*. |
| *Inocybe leptophylla* | In coniferous and mixed forest on nutrient rich soil. |
| *Inocybe maculata* | Mainly with broadleaved trees on nutrient rich soil. |
| *Inocybe mixtilis* | Mostly with coniferous trees but also broadleaved trees. |
| *Inocybe muricellata* | With broadleaved or coniferous trees on nutrient rich, calcareous soil. |
| *Inocybe nitiduscula* | Coniferous forest, sometimes broadleaved forest, on calcareous soil. |
| *Inocybe ochroalba* | Somewhat contradictory accounts, from together with *Pine* on sandy soil to with both coniferous and broadleaved trees on calcereous soil. |
| *Inocybe petiginosa* | With *Fagus* and *Quercus*. |
| *Inocybe phaeocomis* | Somewhat contradictory. With coniferous and broadleaved trees on calcareous soil or in broadleaved, mixed forests or parks. |
| *Inocybe quietiodor* | With broadleaved trees on calcareous, nutrient rich soil. |
| *Inocybe rimosa* | With broadleaved or coniferous trees. |
| *Inocybe soluta* | Mostly in coniferous forest but also broadleaved forest. |
| *Inocybe pseudoasterospora* | In coniferous and/or broadleaved forest on nutrient rich soil. |

E.

| Species | Voucher | Accession # |
|---|---|---|
| *Inocybe acuta* | EL2405 | AM882744 |
| *Inocybe acutella* | EL2304 | AM882930 |
| *Inocybe acutella* | EL5505 | AM882923 |
| *Inocybe acutella* | SJ83045 | AM882922 |
| *Inocybe adaequata* | MR00022 | AM882706 |
| *Inocybe albomarginata* | BJ900831 | AM882908 |
| *Inocybe albomarginata* | TAA185187 | AM882808 |
| *Inocybe alnea* | BJ900827 | AM882717 |
| *Inocybe alpigenes* | EL4005 | AM882910 |
| *Inocybe alpigenes* | EL4105 | AM882751 |
| *Inocybe alpigenes* | EL4905 | AM882750 |
| *Inocybe ambigua* | BJ910730 | AM882800 |
| *Inocybe amethystina* | SG1997 | AM882892 |
| *Inocybe appendiculata* | SJ9205 | AM882806 |
| *Inocybe arthrocystis* | EL4805 | AM882856 |
| *Inocybe asterospora* | MR00015 | AM882897 |
| *Inocybe asterospora* | EL14305 | AM882722 |
| *Inocybe aurea* | EL10905 | AM882740 |
| *Inocybe aurea* | SJ80039 | AM882739 |
| *Inocybe boltonii* | EL8905 | AM882754 |
| *Inocybe boltonii* | BJ920816 | AM882753 |
| *Inocybe bongardii* | EL6104 | AM882943 |
| *Inocybe bongardii* | TAA185132 | AM882942 |
| *Inocybe bongardii* | EL12304 | AM882941 |
| *Inocybe bongardii* | EL7004 | AM882940 |
| *Inocybe calamistrata* | KHL13071 | AM882948 |
| *Inocybe calamistrata* | EL4305 | AM882947 |
| *Inocybe calamistrata* | EL2605 | AM882946 |
| *Inocybe calamistrata* | EL13004 | AM882944 |
| *Inocybe calamistrata* | EL1904 | AM882938 |
| *Inocybe calida* | BJ910802 | AM882900 |
| *Inocybe calida* | TAA185175 | AM882760 |
| *Inocybe calida var. bruneorufa* | SJ78072 | AM882898 |

| Species | Voucher | Accession # |
| --- | --- | --- |
| *Inocybe calospora* | EL9505 | AM882759 |
| *Inocybe calospora* | BJ840909 | AM882758 |
| *Inocybe castanea* | SJ03013 | AM882719 |
| *Inocybe castanea* | EL904 | AM882718 |
| *Inocybe cervicolor* | EL7703 | AM882945 |
| *Inocybe cervicolor* | SJ04024 | AM882939 |
| *Inocybe cervicolor* | EL2799 | AM882937 |
| *Inocybe cookei* | EL10904 | AM882956 |
| *Inocybe cookei* | EL7305 | AM882955 |
| *Inocybe cookei* | MR00035 | AM882954 |
| *Inocybe cookei* | EL70A03 | AM882953 |
| *Inocybe cookei* | EL10404 | AM882952 |
| *Inocybe corydalina* | EL12404 | AM882736 |
| *Inocybe cryprocystis* | BJ900914 | AM882906 |
| *Inocybe curvipes* | EL9805 | AM882814 |
| *Inocybe curvipes* | EL6703 | AM882813 |
| *Inocybe decipiens* | TAA185128 | AM882830 |
| *Inocybe devonensis* | TAA17205 | AM882826 |
| *Inocybe diabolica* | JV5712 | AM882903 |
| *Inocybe dulcamara* | EL7804 | AM882865 |
| *Inocybe dulcamara* | EL6905 | AM882861 |
| *Inocybe dulcamara* | EL5905 | AM882863 |
| *Inocybe dulcamara* | EL304 | AM882859 |
| *Inocybe dulcamara* | EL3305 | AM882854 |
| *Inocybe dunensis* | TAA171822 | AM882829 |
| *Inocybe dunensis* | SJ77325 | AM882828 |
| *Inocybe egenula* | EL4605 | AM882714 |
| *Inocybe erinaceomorpha* | EL12805 | AM882735 |
| *Inocybe erinaceomorpha* | SJ20010 | AM882734 |
| *Inocybe erubescens* | KGN980714 | AM882951 |
| *Inocybe erubescens* | TAA185164 | AM882950 |
| *Inocybe erubescens* | BJ910707 | AM882949 |
| *Inocybe fibrosa* | EL2599 | AM882846 |
| *Inocybe fibrosoides* | SS2990 | AM882827 |

| Species | Voucher | Accession # |
|---|---|---|
| *Inocybe flavella* | EL11805 | AM882782 |
| *Inocybe flavella* | SJ04005 | AM882779 |
| *Inocybe flavella* | EL13705 | AM882776 |
| *Inocybe flavella* | LAS89030 | AM882775 |
| *Inocybe flavella* | BJ920829 | AM882774 |
| *Inocybe flavella* | EL9004 | AM882773 |
| *Inocybe flocculosa* | EL10504 | AM882893 |
| *Inocybe flocculosa* | EL1004 | AM882891 |
| *Inocybe flocculosa* | EL9504 | AM882890 |
| *Inocybe flocculosa* | EL3504 | AM882889 |
| *Inocybe fraudans* | EL6405 | AM882733 |
| *Inocybe fraudans* | SJ82030 | AM882732 |
| *Inocybe fraudans* | EL11604 | AM882731 |
| *Inocybe fraudans* | EL15105 | AM882730 |
| *Inocybe fraudans* | EL12304 | AM882729 |
| *Inocybe fulvipes* | EL3705 | AM882858 |
| *Inocybe fuscidula* | BJ911005 | AM882888 |
| *Inocybe fuscidula* | EL504 | AM882887 |
| *Inocybe fuscidula* | EL9505 | AM882886 |
| *Inocybe fuscidula* | RP900608 | AM882884 |
| *Inocybe fuscidula* | EL15903 | AM882883 |
| *Inocybe fuscidula* | EL1604 | AM882842 |
| *Inocybe fuscidula var. bisporigera* | EL14405 | AM882894 |
| *Inocybe geophylla* | EL8003 | AM882877 |
| *Inocybe geophylla* | EL9005 | AM882870 |
| *Inocybe geophylla var. lilacina* | EL7903 | AM882876 |
| *Inocybe geophylla var. lilacina* | EL12605 | AM882875 |
| *Inocybe geophylla var. lilacina* | EL11304 | AM882874 |
| *Inocybe geophylla var. lilacina* | EL9205 | AM882873 |
| *Inocybe geophylla var. lilacina* | EL5004 | AM882869 |
| *Inocybe giacomi* | EL4205 | AM882745 |
| *Inocybe giacomi* | EL2805 | AM882743 |
| *Inocybe giacomi* | EL3105 | AM882742 |

| Species | Voucher | Accession # |
|---|---|---|
| *Inocybe glabripes* | TAA145067 | AM882902 |
| *Inocybe glabripes* | BJ940922 | AM882807 |
| *Inocybe glabripes* | EL10805 | AM882794 |
| *Inocybe godeyi* | MJ3992 | AM882804 |
| *Inocybe godeyi* | EL8404 | AM882803 |
| *Inocybe grammata* | SJ000903 | AM882841 |
| *Inocybe griseolilacina* | EL6704 | AM882728 |
| *Inocybe griseoscabrosa* | BJ900922 | AM882791 |
| *Inocybe griseoscabrosa* | SJ93026 | AM882790 |
| *Inocybe gymnocarpa* | SJ980707 | AM882866 |
| *Inocybe haemacta* | SJ88062 | AM882737 |
| *Inocybe hirculus* | SJ020820 | AM882723 |
| *Inocybe hirtella* | MR00025 | AM882934 |
| *Inocybe hirtella* | EL13505 | AM882933 |
| *Inocybe hirtella var. bispora* | EL12505 | AM882932 |
| *Inocybe hystrix* | EL7604 | AM882812 |
| *Inocybe hystrix* | MR00048 | AM882811 |
| *Inocybe hystrix* | SJ020824 | AM882810 |
| *Inocybe impexa* | EL15605 | AM882815 |
| *Inocybe inodora* | DO870712 | AM882901 |
| *Inocybe inodora* | EL2405 | AM882834 |
| *Inocybe jacobi* | SJ980727 | AM882710 |
| *Inocybe lacera* | TAA128324 | AM882860 |
| *Inocybe lacera* | EL2104 | AM882823 |
| *Inocybe lacera* | EL39A04 | AM882822 |
| *Inocybe lacera* | EL5705 | AM882820 |
| *Inocybe lacera* | EL505 | AM882819 |
| *Inocybe lacera* | EL805 | AM882818 |
| *Inocybe lacera* | EL5305 | AM882816 |
| *Inocybe lacera var. rhacodes* | EL5605 | AM882817 |
| *Inocybe langei* | EL7705 | AM882919 |
| *Inocybe langei* | EL1203 | AM882918 |
| *Inocybe lanuginosa var. longicystis* | SJ910726 | AM882845 |

| Species | Voucher | Accession # |
|---|---|---|
| *Inocybe lanuginosa* | RM5119 | AM882843 |
| *Inocybe lanuginosa* | SJ88059 | AM882824 |
| *Inocybe lanuginosa* | F50698 | AM882788 |
| *Inocybe leiocephala* | EL7504 | AM882793 |
| *Inocybe leptocystis* | SJ96002 | AM882801 |
| *Inocybe leptophylla* | BJ920801 | AM882787 |
| *Inocybe leucoblema* | SEB9709 | AM882857 |
| *Inocybe leucoblema* | EL5005 | AM882855 |
| *Inocybe maculata* | EL12604 | AM882964 |
| *Inocybe maculata* | EL5803 | AM882963 |
| *Inocybe maculata* | EL7803 | AM882962 |
| *Inocybe maculata* | EL7405 | AM882959 |
| *Inocybe maculata* | MR00020 | AM882958 |
| *Inocybe maculata* | EL12104 | AM882957 |
| *Inocybe malenconii* | BJ900815 | AM882867 |
| *Inocybe malenconii* | SJ030822 | AM882862 |
| *Inocybe maritima* | TAA172127 | AM882821 |
| *Inocybe melanopoda* | JV4986 | AM882727 |
| *Inocybe melanopus* | TAA185135 | AM882726 |
| *Inocybe melanopus* | BJ920904 | AM882725 |
| *Inocybe mimica* | TK2004114 | AM882781 |
| *Inocybe mixtilis* | BJ920916 | AM882840 |
| *Inocybe mixtilis* | EL5904 | AM882839 |
| *Inocybe mixtilis* | EL13104 | AM882838 |
| *Inocybe mixtilis* | EL2604 | AM882837 |
| *Inocybe mixtilis* | EL8904 | AM882836 |
| *Inocybe mixtilis* | EL9604 | AM882835 |
| *Inocybe muricellata* | BJKGN980720 | AM882917 |
| *Inocybe muricellata* | KGN980725 | AM882916 |
| *Inocybe muricellata* | EL3704 | AM882915 |
| *Inocybe muricellata* | EL6705 | AM882909 |
| *Inocybe napipes* | EL7005 | AM882927 |
| *Inocybe napipes* | EL6105 | AM882926 |
| *Inocybe napipes* | EL3204 | AM882925 |

| Species | Voucher | Accession # |
|---|---|---|
| *Inocybe napipes* | EL14105 | AM882924 |
| *Inocybe nematoloma* | EL10105 | AM882713 |
| *Inocybe nematoloma* | BJ940812 | AM882712 |
| *Inocybe nitidiuscula* | EL11605 | AM882913 |
| *Inocybe nitidiuscula* | EL16205 | AM882912 |
| *Inocybe nitidiuscula* | EL1304 | AM882911 |
| *Inocybe nitidiuscula* | EL11804 | AM882847 |
| *Inocybe oblectabilis* | MB980915 | AM882833 |
| *Inocybe oblectabilis* | Blom9909 | AM882832 |
| *Inocybe oblectabilis* | BJ920908 | AM882831 |
| *Inocybe obscurobadia* | SJ03008 | AM882802 |
| *Inocybe obsoleta* | BJ890915 | AM882770 |
| *Inocybe obsoleta* | EL1704 | AM882769 |
| *Inocybe ochroalba* | EL5704 | AM882882 |
| *Inocybe ochroalba* | MR00031 | AM882881 |
| *Inocybe ochroalba* | EL8604 | AM882880 |
| *Inocybe ochroalba* | EL4704 | AM882879 |
| *Inocybe ochroalba* | EL4804 | AM882878 |
| *Inocybe pelargonium* | TK04063 | AM882936 |
| *Inocybe perlata* | BJ940922 | AM882772 |
| *Inocybe perlata* | EL7404 | AM882771 |
| *Inocybe petiginos* | EL6304 | AM882708 |
| *Inocybe petiginosa* | BJ911004 | AM882709 |
| *Inocybe petiginosa* | EL2004 | AM882707 |
| *Inocybe phaeocomis* | EL10704 | AM882853 |
| *Inocybe phaeocomis* | SJ05048 | AM882852 |
| *Inocybe phaeocomis* | EL13404 | AM882851 |
| *Inocybe phaeocomis* | EL6205 | AM882850 |
| *Inocybe phaeocomis* | EL2504 | AM882849 |
| *Inocybe phaeocomis* | EL204 | AM882848 |
| *Inocybe phaeodisca* | BJ940823 | AM882905 |
| *Inocybe posterula* | EBJ051120 | AM882868 |
| *Inocybe praetervisa* | EL12904 | AM882720 |
| *Inocybe pseudoasterospora* | SJ88043 | AM882921 |

| Species | Voucher | Accession # |
|---|---|---|
| *Inocybe pseudoasterospora* | SJ92056 | AM882920 |
| *Inocybe whitei* | EL15905 | AM882872 |
| *Inocybe whitei* | BJ940821 | AM882871 |
| *Inocybe putilla* | RP010808 | AM882752 |
| *Inocybe quietiodor* | LAS94023 | AM882961 |
| *Inocybe quietiodor* | EL11504 | AM882960 |
| *Inocybe relicina* | EL3603 | AM882795 |
| *Inocybe rennyi* | SG0306 | AM882741 |
| *Inocybe rennyi* | JV900818 | AM882716 |
| *Inocybe rennyi* | SJ88034 | AM882715 |
| *Inocybe rimosa* | TK97156 | AM882844 |
| *Inocybe rimosa* | EL7104 | AM882786 |
| *Inocybe rimosa* | SJ81051 | AM882777 |
| *Inocybe rimosa* | EL12704 | AM882768 |
| *Inocybe rimosa* | TAA185165 | AM882767 |
| *Inocybe rimosa* | TAA185135 | AM882766 |
| *Inocybe rimosa* | EL6605 | AM882765 |
| *Inocybe rimosa* | EL5105 | AM882764 |
| *Inocybe rimosa* | SJ04007 | AM882763 |
| *Inocybe rimosa* | EL7505 | AM882762 |
| *Inocybe rimosa* | EL10204 | AM882761 |
| *Inocybe salicis* | EL7105 | AM882724 |
| *Inocybe sambucina* | SJ01002 | AM882757 |
| *Inocybe sapinea* | BJ910825 | AM882797 |
| *Inocybe sapinea* | EL8105 | AM882796 |
| *Inocybe sindonia* | EL9105 | AM882896 |
| *Inocybe sindonia* | EL8504 | AM882895 |
| *Inocybe soluta* | SJ86040 | AM882904 |
| *Inocybe soluta* | BJ840724 | AM882756 |
| *Inocybe soluta* | EL2904 | AM882755 |
| *Inocybe squamata* | SJ92010 | AM882785 |
| *Inocybe squamata* | SJ92017 | AM882784 |
| *Inocybe squamata* | SM92013 | AM882783 |
| *Inocybe squamata* | TK96109 | AM882780 |

| Species | Voucher | Accession # |
|---|---|---|
| *Inocybe squamata* | J85048 | AM882778 |
| *Inocybe stellatospora* | EL3004 | AM882747 |
| *Inocybe stellatospora* | BJ941016 | AM882746 |
| *Inocybe subexilis* | BJ910730 | AM882711 |
| *Inocybe subnudipes* | SJ94024 | AM882907 |
| *Inocybe subnudipes* | BJ920916 | AM882809 |
| *Inocybe subporospora* | RP950618 | AM882931 |
| *Inocybe tenebrosa* | SJ88053 | AM882899 |
| *Inocybe tenebrosa* | RGC97087 | AM882792 |
| *Inocybe teraturgus* | SJ79017 | AM882789 |
| *Inocybe terrigena* | EL11704 | AM882864 |
| *Inocybe tetragonospora* | EL4505 | AM882749 |
| *Inocybe tetragonospora* | EL1505 | AM882748 |
| *Inocybe tjallingiorum* | BJ940912 | AM882805 |
| *Inocybe tricolor* | SJ05011 | AM882738 |
| *Inocybe umbratica* | BJ920804 | AM882799 |
| *Inocybe umbratica* | SJ03020 | AM882798 |
| *Inocybe umbrina* | EL14805 | AM882929 |
| *Inocybe umbrina* | EL7503 | AM882928 |
| *Inocybe vulpinella* | EL000610 | AM882825 |
| *Inocybe xanthomelas* | SJ95027 | AM882721 |
| *Inocybe cf. acuta* | EL13405 | AM882988 |
| *Inocybe cf. boltonii* | EL7403 | AM882965 |
| *Inocybe cf. flocculosa* | EL10605 | AM882992 |
| *Inocybe cf. flocculosa* | EL5804 | AM882969 |
| *Inocybe cf. fuscidula* | EL8805 | AM882993 |
| *Inocybe cf. fuscidula* | EL15305 | AM882935 |
| *Inocybe cf. fuscomarginat* | BJ890718 | AM882972 |
| *Inocybe cf. geophylla* | TK04137 | AM882984 |
| *Inocybe cf. glabrescens* | BJ900831 | AM882973 |
| *Inocybe cf. grammata* | TAA185173 | AM882976 |
| *Inocybe cf. griseovelat* | TAA185125 | AM882975 |
| *Inocybe cf. hirculus* | Gahne0252 | AM882986 |
| *Inocybe cf. microspora* | EL8103 | AM882971 |

| Species | Voucher | Accession # |
|---|---|---|
| *Inocybe cf. leiocephala* | EL3805 | AM882914 |
| *Inocybe cf. oblectabilis* | Guten990923 | AM882979 |
| *Inocybe cf. oreina* | Gahne0296 | AM882980 |
| *Inocybe cf. pelargonium* | BJ910808 | AM882977 |
| *Inocybe cf. phaeodisca* | SJ95012 | AM882978 |
| *Inocybe cf. praetervisa* | EL3505 | AM882989 |
| *Inocybe cf. praetervisa* | SJ84178 | AM882966 |
| *Inocybe cf. pseudoreduca* | EL9804 | AM882970 |
| *Inocybe cf. pseudoreducta* | EL8204 | AM882967 |
| *Inocybe cf. pusio* | SJ91032 | AM882981 |
| *Inocybe cf. putilla* | Thoresso0007021 | AM882982 |
| *Inocybe cf. rennyi* | TAA142894 | AM882985 |
| *Inocybe cf. resneri* | LAS97067 | AM882974 |
| *Inocybe cf. rimosa* | SJ05029 | AM882994 |
| *Inocybe cf. subnudipes* | BJ910809 | AM882983 |
| *Inocybe sp.* | SJ05030 | AM882995 |
| *Inocybe sp.* | EL1705 | AM882991 |
| *Inocybe sp.* | EL6005 | AM882990 |
| *Inocybe sp.* | EL14905 | AM882987 |
| *Inocybe sp.* | EL404 | AM882968 |
| *Crepidotus mollis* | EL4504 | AM882996 |
| *Naucoria submelinoides* | TAA185174 | AM882885 |
